# In-Depth Analysis of the Data from an Interlaboratory Study of Quantitative Non-Target Screening—How Do the Instrumental Methods Compare?

**DOI:** 10.3390/molecules31050875

**Published:** 2026-03-06

**Authors:** Louise Malm, Nikiforos Alygizakis, Reza Aalizadeh, Anneli Kruve

**Affiliations:** 1 Department of Chemistry, Stockholm University, Svante Arrhenius Väg 16, 114 18 Stockholm, Sweden; louise.malm@su.se; 2 Laboratory of Analytical Chemistry, Department of Chemistry, National and Kapodistrian University of Athens, Panepistimiopolis Zografou, 157 71 Athens, Greece; nalygizakis@chem.uoa.gr; 3 Environmental Institute, Okruẑná 784/42, 972 41 Koš, Slovakia; 4 Department of Environmental Health Sciences, Yale School of Public Health, Yale University, 60 College St., New Haven, CT 065 10, USA; reza.aalizadeh@yale.edu; 5 Department of Environmental Science, Stockholm University, Svante Arrhenius Väg 8, 114 18 Stockholm, Sweden

**Keywords:** interlaboratory comparison, ionization efficiency, liquid chromatography, mass spectrometry, non-target, quantification, response factor

## Abstract

Non-target screening utilizing liquid chromatography–high-resolution mass spectrometry is increasingly employed for the environmental monitoring of contaminants; however, obtaining quantitative results of detected suspected compounds is challenging; different approaches have been suggested. A recent interlaboratory comparison of quantification approaches showed that the machine learning-based approaches leveraging predicted ionization efficiencies outcompete surrogate standard-based approaches, independent of the method used. In this study, we further analyzed data from the interlaboratory comparison to: (1) evaluate whether the prediction errors could be linked to instrument parameters; and (2) investigate the comparability of response factors (RFs) across different datasets to shed light on the limitations of the instrumental method on the predicted ionization efficiency approach. No specific parameters could be linked to systematic effects on the prediction errors; however, the choice of organic modifier and/or additive type influenced the detection of some compounds. Comparable logRFs across datasets were observed when a linear model was used to project the values to the same scale. Nevertheless, the projected logRF scale was compressed for datasets with low similarity to the anchoring dataset. Moreover, compounds with low logRF showed higher variability across the datasets. The data are freely available and can be interrogated in the developed dashboard.

## 1. Introduction

The large number of compounds that can be, and have been, detected in water bodies (rivers, lakes, groundwater reserves, etc.) is concerning and poses adverse risks to both environmental and human health [1,2]. The most common technique to detect these compounds is liquid chromatography–high-resolution mass spectrometry (LC/HRMS), especially for the analysis of environmental contaminants in aqueous samples [3,4,5]. Combined with electrospray ionization (ESI), compounds of a polar to semipolar nature with molecular masses up to 100 000 Da can be analyzed [6], with methods usually screening between 50–1200 *m*/*z*. Thus, with one analysis technique, a relatively large fraction of an environmentally relevant chemical space can be detected. Traditionally, targeted methods are used to monitor the occurrence of contaminants in the environment. However, there are many compounds in daily use that can easily be released and spread to the environment. Therefore, non-targeted screening (NTS) approaches are needed to detect, characterize the risk, and find the source of these organic pollutants [7,8]. NTS, as opposed to targeted methods, aims to unravel all compounds that can be detected with the chosen analysis technique. Although NTS theoretically enables the detection of novel contaminants—so-called “unknown unknowns”—the analysis method, as well as all steps prior to it, e.g., sampling and sample preparation, can influence its sensitivity [6,9]. Currently, library matching and de novo structure generation enable (tentative) structural assignment beyond the compounds with available analytical standards [6], while the importance of the detected compounds depends both on their bioactivity and concentration.

Usually, the quantitative information in target LC/ESI/HRMS is obtained from a calibration graph, i.e., the concentration can be calculated using the slope, also known as the response factor (RF), and the intercept of the calibration graph (Equation (1)):

(1) $$\mathrm{concentration}=\frac{\mathrm{peak}\;\mathrm{area}-\mathrm{intercept}}{\mathrm{RF}}$$

Target quantification requires the analytical standard of the compound(s) of interest and thus limits the quantitative analysis to (well) known and (easily) obtainable compounds. In addition, quantification in ESI is especially problematic due to the diverse responses of compounds [6]. Consequently, the peak area cannot directly indicate the absolute concentration of the compound in the sample; therefore, quantitative non-target LC/ESI/HRMS is hindered. Several approaches to estimate the concentrations without the need of many analytical standards have been developed over the years, from “simpler” approaches using the RF of a surrogate standard [10,11] to machine learning (ML) tools predicting the relative RF or ionization efficiency (*IE*) of the detected compounds [12,13,14,15,16,17,18]. The *IE* depends both on the physicochemical properties of the compound, such as acid-base properties and polarity [12,19,20], as well as on the mobile phase composition (e.g., organic solvent content, pH) at the time of elution [12,21,22,23,24]. Similarly, the RF of a compound depends on both its *IE* as well as on instrumental parameters such as ion source design and ion transport efficiency [8,25].

Our recent work evaluated different quantification methods for NTS and concluded that, across the 37 participating laboratories, *IE* predictions yield the highest accuracy [8]. At the same time, the NTS methods employed in different laboratories differ and no standardized methods have been, nor can they be, established. For example, different organic mobile phase components (commonly, methanol (MeOH) or acetonitrile (MeCN)) as well as mobile phase additives (e.g., formic acid, acetic acid, ammonium format, ammonium acetate) were used by different research groups. Depending on the additive, the pH of the aqueous phase also varied. Even if the same separation mechanism was used for the LC, the columns used were not identical, e.g., the column dimension, stationary phase modifications and particle sizes were different. Moreover, the electrospray ionization source geometries also varied with the MS vendor. The most common designs from the major vendors include in-line spray, offset spray, orthogonal spray, Z-spray or spraying at a 60° angle [26,27]. As a result, the comparability of the NTS methods and their impact on ionization efficiency could be affected and the comparability of the *IE*s or RFs across methods would further impact the quantitative analysis.

In this study, we conduct a deeper analysis of the data from the recent interlaboratory study [8]. The prediction accuracy, expressed as prediction error, is further investigated in relation to instrumental parameters and chemical structures. The analysis is expanded to include comparison of RFs across different datasets (obtained from the different participating laboratories). Due to the wide differences in RFs obtained from different NTS methods and instruments, two methods to project the RFs to the same scale are evaluated. The similarities of projected logRF values, as well as the coherence of projected logRFs across datasets and compounds, are assessed. Lastly, we developed an interactive dashboard (https://norman-data.eu/ILS-dashboard) where the data discussed here can be visualized based on user input.

## 2. Results and Discussion

### 2.1. Prediction Error Analysis

The error in predicted concentration from the collaborative trial was investigated with respect to certain analysis parameters such as the type of organic modifier and additive, analyzer type, gradient length, injection volume, and spray voltage. As seen in the corresponding tabs in the dashboard (under the main tab Quantification error graphs), there were no clear trends indicating that any of the instrumental parameters would have a major systematic effect on the prediction accuracy. Statistical tests (Wilcoxon rank sum test or Friedman test, see Table S1a–g for *p*-values, sample sizes and effect sizes) revealed no statistical evidence in the impact of instrumental parameters, quantification approach, or concentration level in this design or metadata, with two exceptions. First, for the parent–transformation product (TP) approach in the sample with the low concentrated spike, there was a difference between the additive types used (*p* = 0.047), with acid as the additive type yielding a lower mean fold error. Second, the Friedman test showed that there was a difference between the spray voltages in the sample spiked at a high concentration level (*p* = 0.022). Specifically, the post hoc Nemenyi’s test revealed that using a spray voltage below 3.0 kV resulted in a statistically different prediction error compared to using a spray voltage between 3.0 and 3.5 kV (*p* = 0.031). At the same time, more than one parameter changed from dataset to dataset, potentially contributing to the prediction errors.

#### 2.1.1. Gaining Insights into the Prediction Errors with Machine Learning

Since the investigated parameters in most cases co-varied, it was difficult to assess their individual effects on the prediction errors. Therefore, we modelled the prediction errors with ML. Specifically, two random forest (RandFor) models were trained; one to predict log(fold error) and one to predict log(error). Both models were trained using the same input features and with the same ML parameters. As an acceptable predictive power was only observed for the log(error) model, the impact of the features for this model was evaluated with SHAP [28] (Code S2). It was observed that the use of a buffer as an additive yielded underpredictions, while the use of acid as an additive yielded some overpredictions. It was also observed that the buffer resulted in larger absolute error (higher absolute SHAP value). From the SHAP analysis, it appeared that using acid as a mobile phase additive would result in lower prediction error, independent of the quantification approach used. It must be noted that this is an indication, valid only for the chemical space used in this study.

#### 2.1.2. Comparing Prediction Errors and Number of Detected Compounds from the Analysis on the Same Instrument

Four dataset pairs were acquired from the analysis on the same instrument, using either different instrumental methods and/or analysts, different acquisition methods, or analyzing different dilutions of the samples. Thus, it was possible to conduct an in-depth one-on-one comparison. For example, two datasets were analyzed by the same person on the same instrument, using the same column, injection volume, and HRMS parameters, but using a different organic modifier (MeCN or MeOH), additive type (acid or buffer), pH (2.5 or 3.9), and flow rate (300 µL/min or varying). It was observed (Figure S1a) that for parent–TP, structurally similar and RandFor-*IE* approaches, using the MeCN-based method, gave a slightly lower prediction error, while for close eluting and multiple linear regression (MLR)–*IE* approaches, the MeOH-based method provided lower errors; however, a statistical analysis (Wilcoxon rank sum test) showed that there was no significant difference between the concentration errors from these two datasets for any of the approaches (*p* > 0.05).

A difference in the detection frequency between the two datasets was observed: the NTS method based on MeOH detected 72.9% of the calibrants and 85.4% of the suspect compounds, while the other method detected 56.2% and 68.3% of the calibrants and suspect compounds, respectively. This induced the hypothesis that the organic modifier type may affect the detection frequency for the compounds used in this study. Therefore, we compared the detection frequency across all datasets using either MeOH or MeCN as the organic component of the mobile phase. MeOH was used in 18 datasets, of which nine used an acid as additive with a reported pH range of 2.5–4.0 in the aqueous mobile phase. The reported pH range for the nine datasets using a buffer as an additive was wider, from 3.0 to 6.8. Across the datasets using MeOH, 37.5–81.2% (62.5% on average) of the calibrants, and 41.5–90.2% (average 71.3%) of the suspect compounds were detected. MeCN was used in 19 datasets, 18 with an acid as additive (reported pH 2.5–3.0) and one with a buffer as additive (no reported pH). The detection frequency for these datasets was 25.0–77.1% (average 56.2%) for the calibrants and 24.4–87.8% (average 62.3%) for the suspect compounds. Thus, using MeOH as a mobile phase may be beneficial for the detection of the compounds in the sample analyzed in this study. It has been shown previously that the use of MeOH resulted in a higher response, at least for some compounds, compared to MeCN in both positive and negative ESI mode [21,22,29]. For the positive mode, this was linked to longer retention times when using MeOH as an organic modifier, and hence to a higher organic modifier content at the time of elution [29]. The higher sensitivity could be crucial for the detection of compounds that are close to the method limit of detection. While the results here support those from the single-laboratory studies mentioned above, the organic modifier and additive type (and/or concentration) in this study was not systematically varied.

We further compared the detection frequency only for the datasets obtained with MeOH or MeCN in combination with 0.1% formic acid as an additive (with the reported pH ranging from 2.5 to 3). In the eight datasets obtained from the analysis with MeOH and 0.1% formic acid as an additive (reported pH 2.5–2.7), the detection frequency for calibrants ranged from 37.5–79.2% (average 58.6%); for suspect compounds, the detection frequency was 58.5–85.4% (average 71.0%). In the 16 datasets where MeCN and 0.1% formic acid (reported pH 2.5–4.0) were used in the analysis, 31.2–77.1% (average 58.9%) of the calibrants and 24.4–87.8% (average 65.4%) of the suspect compounds were detected. Thus, a larger fraction of suspect compounds was generally detected in analyses utilizing MeOH as the organic modifier. For the calibrants, however, the average detection frequencies were essentially the same for analyses with MeOH and MeCN. This implied that the detection frequency of the compounds used in this study most likely depended on a combination of the organic modifiers, the additive, and the pH of the aqueous phase, as well as on the compound itself. To assess if certain compound classes were preferably detected in any specific mobile phase composition, the compounds were classified using ClassyFire [30]. Then, the detection frequency was calculated for each mobile phase composition, i.e., MeOH–acid, MeOH–buffer, MeCN–acid, and MeCN–buffer, for all ions ([M+H]^+^, [M+Na]^+^, [M+NH_4_]^+^, Table S2). In total, 15 ion species (chemical with respective ion type) were detected in less than 25% of the datasets: ten calibrants and five suspect compounds. However, no detection patterns regarding these were observed for specific compound classes.

Two other datasets were obtained from the same instrument and instrumental method, only the acquisition mode differed: data were either acquired with data-dependent acquisition (DDA) or full-scan acquisition (MS^1^). There was no statistically significant difference between the prediction errors for any of the quantification approaches in these two datasets (Figure S1b). Nevertheless, a difference in the number of detected compounds was observed. In the dataset that used DDA, 50.0% of the calibrants and 58.5% of the suspect compounds were detected, while in the dataset using MS^1^, 62.5% of calibrants and 70.7% of suspect compounds were detected. This is most likely because a higher number of points per peak is obtained for MS^1^ compared to DDA in full scan, while for some compounds, the points per peak when DDA is triggered is too low to yield peaks of acceptable quality.

### 2.2. Response Factor Analysis

Due to the variation in instrumentation used, the resulting peak areas varied by several orders of magnitude across datasets; consequently, the resulting RFs also varied widely. In order to compare RF values, it was necessary to log-transform and project the logRFs to the same scale. For this, a linear model (LM) and a generalized additive model (GAM) were fitted separately to the calibrants, using L38 (measurements from Stockholm University, roughly half of the datasets were acquired using a similar mobile phase) as the anchoring dataset (x-axis). The number of overlapping compounds varied, with a minimum of eight overlapping compounds for one dataset. The performance of the projection methods was evaluated based on the root mean squared errors (RMSEs) of the suspect compounds for each dataset, compared using an *F*-test. This showed that in general, there was no statistical difference between the two projection methods, with the exception of one dataset, namely L20 (Table S3). Therefore, we also compared the sum of squares of residuals (SS_res_) (Equation (2)) and observed that it was lower when using LM in general, and specifically for the significant dataset—possibly indicating that GAM had too much flexibility and was prone to overfitting the calibration data. Furthermore, LM has been widely used to link different datasets and transfer predicted log*IE* values to logRF [14,16]. The results here showed that this approach is valid across a large number of datasets. Therefore, we continued the analysis with the LM-projected logRF values.

The linear regression between logRF values from different instruments and methods assumes that the main factors affecting RF values are common across instruments and methods, e.g., relative ionization efficiency in the ESI. Furthermore, it assumes that other factors, e.g., ion transmission efficiency or the impact of exact ESI conditions used in the methods, impact all compounds similarly or at least to a substantially lower extent. Thus, cases where logRF values do not correlate well between instruments and methods may indicate cases where these assumptions do not hold and instrumental parameters have substantial chemical specific effects that overweigh this on compound intrinsic ionization efficiency.

As seen in tab ‘Response factor analysis > Projected response factors’ in the dashboard, using LM to project the logRFs to the same scale enabled more comparable logRFs across datasets, compared to raw logRF or GAM projected logRF. Some datasets (L1, L16, L29) showed very little improvement, as the raw logRF values were well aligned with the anchoring dataset. Those measurements were carried out using similar mobile phases, HRMS instruments, and settings as L38. On the other hand, L21 used similar mobile phases and HRMS types and settings, but the raw logRF values were systematically shifted compared to the anchoring dataset. Additionally, L17, which used very different LC/HRMS methods (e.g., different organic modifier, different mobile phase additive, different HRMS vendor and mass analyzer), still showed well aligned raw logRFs with L38. Thus, while similar LC/HRMS methods are likely to yield comparable logRF values after LM transformation, this is not always true and different methods may also yield highly agreeing results. Nevertheless, direct comparison of the raw logRF values should be avoided between instruments, samples and methods, preferably even across time. It must be noted that the processing software used also has an effect on the obtained peak areas, and therefore also on the logRF values [31]. A change in the logRF values was observed for many of the compounds and datasets in the original study, ref. [8] depending on the types of peak areas used (reported or reprocessed).

**Figure 1:**
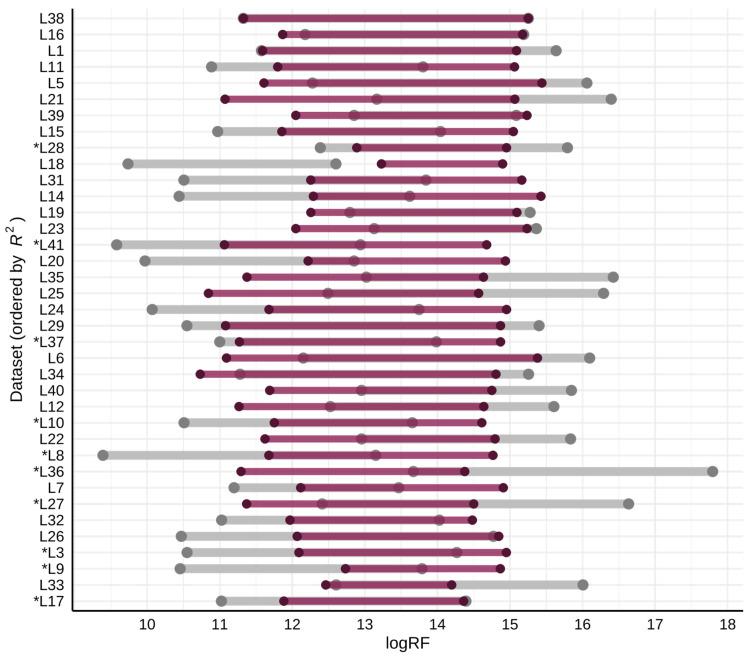
The logRF range for each dataset ordered by the *R*^2^ value (Pearson correlation between projected logRF and the logRF of the anchoring dataset). The raw logRF range is shown in grey (lighter color) and the projected range in purple (darker color). The asterisk indicates the datasets originating from a method using a buffer as the mobile phase additive.

For approximately half of the datasets (20 out of 36), the projection resulted in a narrower logRF range than originally observed (Figure 1). The raw logRF ranges varied from 2.3 to 5.7 orders of magnitude, depending on the dataset, while the projected logRF ranges varied between 1.7 and 5.1 orders of magnitude. In the anchoring dataset, the logRFs ranged nearly four orders of magnitude (logRF range 11.3–15.3). For a few datasets (L18, L33, L28, and L9) it was seen that the projected logRF range was very narrow, ranging only by around two orders of magnitude or less (Figure 1, Table 1). One hypothesis was that if the correlation measured as a squared correlation coefficient (*R*^2^) value (Pearson correlation between projected logRF and the logRF of the anchoring dataset) was poor, this would lead to a more compressed projected logRF range. To check this, the *R*^2^ was plotted against the range ratio, defined as the raw logRF range/projected logRF range. A weak negative trend was observed (Figure S2), as eight out of the 11 datasets with the most compressed scales (range ratio >1.2) were observed for datasets where the *R*^2^ was also below 0.5. Nevertheless, two of the four datasets with the narrowest projected logRF range (L18 and L28, Table 1) had *R*^2^ values over 0.65. Moreover, seven datasets had lower *R*^2^ values (<0.5), while they still provided very similar or even larger logRF ranges than before; five datasets with *R*^2^ < 0.5 had a range ratio of 0.8–1.0, while two datasets had an *R*^2^ < 0.5 and a range ratio of 1.1–1.2.

For L18 and L9, the projected ranges were also shifted towards higher values (projected logRF range: 13.2–14.9 for L18 and 12.7–14.9 for L9), indicating that only well ionizing compounds were detected; however, the original range was around three orders of magnitude for these datasets (raw logRF range: 9.7–12.6 for L18 and 10.5–13.8 for L9). Furthermore, the ten compounds with the lowest average projected logRFs were not detected by L18 or L9, strengthening the abovementioned indication. The majority of these compounds were spiked into the samples at mid to high concentrations (4.0–8900 nM) compared to all compounds (0.85–8900 nM, Table S4). It is important to note, however, that just because a compound has a low detection frequency, it does not mean that it has a low log*IE*. Other reasons for not detecting the compound include co-elution problems, matrix effects, instrument specific issues, or data-processing parameters. Nevertheless, the two main reasons for a narrow logRF range seem to be poor correlation with the anchoring dataset and an inability to detect poorly ionizing compounds. Therefore, we recommend using caution when interpreting projected logRF if the *R*^2^ value is below 0.5, especially when drawing quantitative conclusions.

#### 2.2.1. Similarity of Projected logRF

The similarities of the projected logRFs were examined using Pearson and Spearman correlations, together with a heatmap across the datasets. The two correlation coefficients (*R*^2^) agreed very well across datasets (Table S5) and resulted in very similar clustering (Figure S3, Table S6), with only four datasets clustered differently using Pearson vs. Spearman *R*^2^. Since the relationships were expected to be linear, the data were continuous, and the number of outliers were minimal, we focused only on the Pearson correlation and clusters here.

The highest correlation was found between L22 and L40 (*R*^2^ = 0.991), L23 and L39 (*R*^2^ = 0.984), and L18 and L31 (*R*^2^ = 0.979). For the first two pairs, this is hardly surprising, as the analysis was performed using the same instrument and settings. The only difference was that in the first case, different acquisition modes were used to obtain the data; in the second case, the samples were analyzed in different dilutions. In the third case, however, the two datasets originated from measurements from different laboratories, using different instruments, columns, and instrumental settings. What was similar between datasets L18 and L31 were the mobile phases; both analyses were performed using 0.1% formic acid in water and in MeCN. Interestingly, two other dataset pairs were also acquired on the same instrument, namely L1 and L38, and L10 and L14; however, different methods were used. For the first pair, both the LC and HRMS parameters were relatively similar; consequently, these two datasets showed high correlation (*R*^2^ = 0.901). On the other hand, for the pair L10 and L14, although the data were acquired on the same instrument in the same laboratory, the analytical methods leveraged different mobile phase compositions, pHs, additive types, and flow rates, while the column and HRMS parameters were kept the same. For this pair, the correlation was weak (*R*^2^ = 0.440). This is expected, as mobile phase parameters have been previously shown to have a large effect of ionization efficiency [23,24].

Looking at the heatmap (Figure S3a), we could see three relatively clear clusters of datasets: L12, L22, L23, L25, L35, L39, L40 formed one cluster; another cluster was formed with L1, L11, L16, L28, L38; and one final cluster was formed with the datasets L3, L9, L10, L17, L27, L37, L41. In the first cluster, all datasets utilized similar columns (C18), organic modifiers and additives (MeOH or MeOH with isopropanol, and formic acid), all were collected on an Orbitrap instrument, and used a similar spray voltage (3.5–4 kV). However, two additional datasets (L7 and L34) were acquired with similar parameters, but were not clustered together with these datasets. One of these used a slightly lower concentration of formic acid as an additive (0.05% instead of 0.1%) and yielded a higher reported pH (pH 4 instead of 2.7 or lower). The *R*^2^ within the cluster ranged from 0.654 for L22 vs. L25, to 0.991 for L22 vs. L40. For the two other datasets, the *R*^2^ ranged between 0.416 (L7 vs. L40) and 0.679 (L7 vs. L39).

For the second cluster (L1, L11, L16, L28, L38), the common parameters were that the organic modifier was MeCN, and a similar reported pH (pH 3 or lower) and spray voltage (3.4–3.7 kV) were used. Other parameters, such as column chemistry (reversed phase or hydrophilic interaction liquid chromatography (HILIC)), mobile phase additive (acid or buffer), flow rate (fixed or varying), and analyzer type (Orbitrap or time-of-flight (ToF)), varied from lab to lab. On the other hand, five other datasets also leveraged the same organic modifier, pH and spray voltage, but did not cluster together with the other datasets. Thus, the instrumental reasons leading to the cluster remain unclear.

In the last cluster (L3, L9, L10, L17, L27, L37, L41), all datasets used a buffer as the mobile phase additive; however, varying buffer types and concentrations were used, and the reported pHs of the aqueous mobile phases varied widely (pH 3–6.8). Still, most datasets utilizing buffer as an additive were clustered together with three exceptions: L5, L28, and L36. Similar to the previous cluster, the reasons for these three datasets clustering separately remains unclear from the reported metadata.

From the clustering analysis, we could also see that for one dataset (L33), the projected logRF values were dissimilar to the projected logRFs (*R*^2^ ranging from <0.001 to 0.468) for all datasets. In this case, the data were acquired from nano-LC/ESI/HRMS analysis, which is expected to enhance the *IE* [32], and, therefore, the logRF. On the other hand, it was found that the increased logRF for nanospray seems to be dependent on the size and hydrophobicity of the compounds, and is thus not applicable for all compounds [33]. Moreover, some studies have shown that all compounds should have the same, or a very similar, response in nanospray [34,35], which is not what we observed here for this dataset. As seen in Figure 1, the raw logRF for this dataset ranged over nearly four orders of magnitude. The projected logRF range for L33 was only around two orders of magnitude, the second narrowest of all datasets, with projected logRF values approximately in the middle of the overall range across all datasets.

#### 2.2.2. Similarity of Trends in logRF and Retention Time

Recently, we separately evaluated the retention time orders (RTO) from the same interlaboratory comparison data used here [36]. The clusters observed, based on the RTO and logRF, were generally inconsistent, with one exception. The third cluster discussed above, where all methods utilized a buffer as additive, was also observed when comparing the similarity of RTOs. In the logRF cluster, however, one additional dataset was included in the “buffer” cluster that was not included in the RTO “buffer” cluster. The dataset that utilized nanospray was clustered together with other datasets when comparing RTOs, indicating that the use of nanospray has no or a minor effect on the retention time (RT) or RTO, but a larger effect on the logRF. This is, however, expected, if similar mobile phases and stationary phases are used. Moreover, the dataset that combined a reversed phase and HILIC had, not surprisingly, a large effect on the RTOs; this dataset was omitted in the clustering analysis in the previous paper. Here, this dataset is in one of the clearest clusters described above. Thus, it is clear that the RT and logRF depend on the LC parameters in different ways, which could explain the large prediction errors observed for the closest eluting standard-based quantification approach [8].

#### 2.2.3. Consistency Across Datasets and Compounds

Ideally, projected logRF values would be stable across different datasets, and show little variation depending on instrument and instrumental parameters. This can be visualized with a line plot of the projected logRF values for all compounds across the datasets. As seen in tab ‘Response factor analysis > logRF across datasets’ in the dashboard, the projected logRFs (thicker lines) showed less variation than the raw logRFs (thinner lines). As including too many compounds at the same time makes the plot hard to interpret, we have limited the number of compounds one can include in the plot at the same time. Unfortunately, this makes it difficult to assess which compounds and/or datasets, if any, display more or less coherent projected logRF values. In order to still gain insights to the compound and dataset dependent variations, the sum of squares of difference of dataset-specific logRF and mean logRF (Equation (3)) was determined across both datasets and compounds. The SS varied less across the datasets than across the compounds; the difference of maximum and minimum SS was 0.29 and 0.81 across datasets and compounds, respectively. It was hypothesized that the variation between datasets could be explained by the different injection volumes utilized in the methods. Therefore, the injection volume for the methods used in the three datasets with the highest and lowest SS_data_ (Table 2) was compared. However, very similar injection volumes were used, and, as such, the injection volume used could not be linked to the observed SS_data_ variation.

As for the stability across compounds (Table 3), the two with the highest and two with the lowest SS (sucralose, saccharin, avermectin B1a and reserpine, all protonated species) were detected by very few methods, which may have resulted from the low *IE* in positive ESI. The average projected logRFs were 11.5, 11.9, 13.3 and 14.1 for sucralose, saccharin, avermectin B1a and reserpine, respectively. Comparing these values to the average projected logRF range across all compounds, 11.5–15.0, we could see that sucralose and saccharin were on the lower side of the logRF range. The average logRF of avermectin B1a was approximately in the middle of all studied compounds, and reserpine yielded a rather high average logRF. For sucralose and saccharin, this is expected from the chemical structures. Sucralose does not contain any basic nitrogen atoms, which are the preferred protonation site in the solution phase; however, its oxygen-rich structure likely promotes the formation of sodium adducts [37]. It is also a very polar compound, and thus has low surface activity, contributing to a low *IE*. In fact, both the sodium and ammonium adducts were detected to a much larger extent than the protonated ion for this compound; in 29 and 26 datasets, sodium and ammonium adduct were detected, respectively. Similar to sucralose, avermectin B1a only contains carbon, hydrogen, and oxygen atoms; thus, adduct formation is likely in positive ESI. This is probably the reason why the protonated species was only detected in four datasets. Saccharin was only detected in six datasets and we have previously determined it as having a low *IE* [14,16]. Reserpine was also detected in only two datasets, but the low number of detections could not be related to low *IE*. In fact, it was originally detected in 28 datasets; however, many of these detections did not meet the quality assurance criteria: either because it was only detected in the high concentrated samples (three datasets) or because the peak area ratio of high and low concentration samples was above 20 (ranging from 23 and 261). The peak areas in the high concentrated HPLC water (considered matrix-free) were almost always larger than in the other matrices (tap- and lake-water). This might indicate matrix effects (severely) suppressing the signal from reserpine in the tap- and lake-water. Since the peak area ratios were above 20 in most datasets for the HPLC water samples (ten being the theoretical value), this also indicates a severely nonlinear peak area to concentration relationship in the studied concentration range.

At the same concentration, compounds with low *IE* are harder to detect and distinguish from the background; therefore, it is reasonable to assume that the logRFs of compounds with low *IE* would vary more across different datasets. As discussed above, sucralose and saccharin have low *IE* for the protonated species and thus were only detected in a few datasets. On the other hand, avermectin B1a and reserpine were only detected in four and two datasets, respectively, but had very low SS. Thus, extreme SS can be expected for compounds with very few datapoints (in this case, observations). A weak (but statistically significant) negative correlation between the SS_comp_ and average projected logRF was observed (Figure S4). This indicates that compounds with lower projected logRFs, in general, yield higher SS_comp_, and hence lower agreement, across datasets.

**Figure 2:**
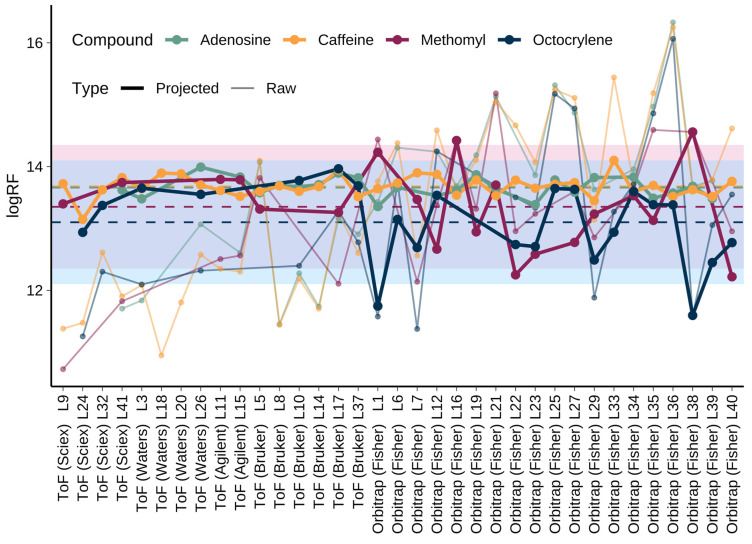
The two compounds with the highest (purple and blue, darker colored dots) and lowest (green and orange, lighter colored dots) SS detected in more than ten datasets. The lines connecting the dots were added to guide the reader to follow the variation in logRF across the datasets. The dashed lines show the average projected logRF for each of the four compounds: 13.66 for adenosine; 13.68 for caffeine; 13.35 for methomyl; and 13.10 for octocrylene. The blue and pink boxes highlight the area that is ± one log unit from the average projected logRF of methomyl and octocrylene, respectively. For methomyl, the projected logRF is outside this range in four datasets (L16, L22, L38, and L40), and for octocrylene in two datasets (L1 and L38). The thicker dots/lines correspond to the projected logRF while the thinner dots/lines show the raw logRF.

Due to the possibly biased SS for the two compounds with the highest and lowest SS, we analyzed the compounds with the third and the fourth highest and lowest SS (Table 3). Methomyl, octocrylene, caffeine, and adenosine were all detected in more datasets; thus, their SS are more reliable. These compounds showed either very stable or quite varying projected logRF across the datasets (Figure 2). For methomyl and octocrylene, it was also observed that the projected logRF variation was mostly appearing for orbitrap instruments; for ToF instruments, very little variation was observed, independent of vendor. Based on single-laboratory measurements, the reproducibility of logRF is expected to be around 0.3 log-units [20]; a wider range is expected for interlaboratory studies. Hence, here we set the limit at ± one log-unit. In a few datasets, the projected logRF value fell outside the mean(logRF) ± one log-unit range. Namely, for methomyl, this occurred in four datasets (L16, L22, L38, and L40); for octocrylene, it occurred in two datasets (L1, and L38). For the orbitrap datasets, and particularly the abovementioned datasets, the mobile phases used were very similar with respect to the reported pH. As a comparison, the reported pH range of aqueous phases in ToF instruments was from 2.5 to 6.75, as the majority of datasets utilizing a buffer as additive also used ToF instruments. Thus, there were no indications that the pH would influence the repeatability of either methomyl (strongest basic p*K*_a_ −0.31) or octocrylene (p*K*_a_ not applicable).

## 3. Materials and Methods

### 3.1. Data

The data used in this study were obtained from an interlaboratory study comparing five quantification approaches used in LC/ESI/HRMS NTS [8]. The experimental details can be found in the previous publication [8]. HPLC water (Honeywell Riedel-de-Haën, Seelze, Germany), tap water, and lake water, spiked with 45 environmental contaminants (suspects), were analyzed with 41 different NTS methods by 37 laboratories, along with standard mixes: HPLC water spiked with 41 compounds at six known concentrations (calibrants). The spiked concentrations were estimated using five quantification approaches, which were evaluated mainly based on the prediction accuracy. In the original study, results are presented as received from the participants, but also as reprocessed raw data. Here, we only use the reprocessed results set, to minimize variation stemming from processing software or the person evaluating the data. In brief, the raw data were reprocessed using an in-house workflow from *patRoon* (v. 2.2.0) [38] and manual inspection of the peaks. The compounds and corresponding peaks were assessed based on their chemical stability in water (assessed over a three-month period), linearity (RFs of calibration curves and peak area ratios between samples spiked with high and low concentrations) and RT. Peaks that did not fulfill the criteria were considered non-detected; the rest of the peaks were normalized to atrazine-d_5_. Due to the unavailability of some raw data, datasets L4, L13 and L30 were excluded from the results. In addition, dataset L2 was excluded as peaks were only detected for the tap-water sample spiked at a lower concentration level in the reprocessing workflow.

Note that in the original study, the peak area ratio between the high and low concentrated samples were used to assess the quality and reliability of detected peaks. The reanalysis of the data performed in this study revealed a minor mistake in the original study: instead of removing the compounds detected only in the lower concentration level, they were kept in the analysis. Here, however, these compounds, along with those only detected in high concentrated samples, have been removed (for motivation, see SI S1). We assessed the impact of including the datapoints from low concentrated samples in the original analysis and found it to be negligible or minor (no statistical tests were performed). A brief comparison of the data can be found in SI S2. Moreover, one dataset was obtained from the analysis of 10× diluted samples; this dilution factor was originally not accounted for. This, however, had no effect on the overall conclusions drawn in the previous study (see SI S3 for a small comparison of the effects).

### 3.2. Statistical Tests of Quantification Results

The prediction fold errors were evaluated based on the following analysis parameters used in the analyses: organic modifier (MeCN or MeOH), mobile phase additive type (acid or buffer), injection volume (<10 µL, 10–25 µL or >25 µL), gradient length (≤25 min or >25 min), analyzer type (Orbitrap or ToF), and spray voltage (<3.0 kV, 3.0–3.5 kV or >3.5 kV). The evaluation was based on visual analysis of the overlap of the interquartile range of the box plots and by statistical tests; the Wilcoxon rank sum test was used for the parameters with two options, and the Friedman test (based on median fold error) was used for the parameters with three options (*p* < 0.05). For the Wilcoxon rank sum test, the adjusted *p*-values were used (Bonferroni correction) to account for multiple testing. Both statistical tests were performed in R using *wilcox_test()* from *rstatix* (v. 0.7.2) [39] package and *friedmanTest()* from *PMCMRplus* (v. 1.9.12) [40] package. The effect sizes for the Wilcoxon rank sum test were obtained from the function *wilcox_effsize()* from *rstatix* [39]. If the Friedman test indicated statistical significance, it was followed up with Nemenyi’s all-pairs comparisons test (*frdAllPairsNemenyiTest()* from *PMCMRplus* [40]). The code for the statistical tests is available in the Supplementary Materials (Code S1).

### 3.3. Machine Learning for Deeper Insight into Prediction Errors

To gain further insights into how the instrumental parameters influenced the prediction errors, two RandFor models were trained to predict either log(fold error) or log(error). Only the HPLC-water sample spiked at a high concentration was considered for the ML. The modelling was conducted in Python (v. 3.12.12) [41] using *GroupShuffleSplit*, *RandomForestRegressor* and *LabelEncoder* from *scikit-learn* (v. 1.8.0) [42]. The compound name, quantification approach, analyzer type, mobile phase additive type, organic modifier, injection volume, spray voltage and gradient length were used as input for the models. The input values were encoded using *LabelEncoder*, and the data were split into a training and test set (80:20 ratio), ensuring that one dataset ended up either in the training or test set using *GroupShuffleSplit*. The RandFor models were trained with 100 trees (n_estimators = 100) and an unlimited tree depth (max_depth = none). The minimum samples to split were set to two (min_samples_split = 2), the minimum samples per leaf was set to one (min_samples_leaf = 1), and random state was set to 42 (random_state = 42). The models were interpreted using SHAP (v. 0.50.0) [28]; data manipulation and visualization were conducted with pandas (v. 2.3.3) [43], NumPy (v. 2.2.6) [44], and Matplotlib (v. 3.10.8) [45]. Claude AI, using Sonnet 4.5, was used to help write the code. The code is available in Code S2.

### 3.4. Determination and Comparison of Response Factors Across Datasets

The logRFs for the calibrants in all datasets were determined as the logarithm of the slope of the linear regression, and for suspect compounds (only in HPLC water) as the average of the two peak areas to the spiked concentration ratios. The logRF values from all the datasets were projected to the scale of L38 (measurements from Stockholm University). These data were chosen as they are already linked to the widest log*IE* collection and are also used to continuously anchor new compounds to this collection [16]. Moreover, these data cover a wide logRF range (close to four orders of magnitude) and were acquired using a mobile phase composition (in terms of organic solvent and additive) comparable to that employed in the majority of the other datasets (18 of 37 datasets). For the projection, an LM and a GAM were fitted separately to the calibrants in R (v. 4.4.1) [46] and applied for suspect compounds. The *lm()* function from *stats* (v. 4.4.1) package and the *gam()* function from *mgcv* (v. 1.9-1) [47] package were used. The *gam()* function used smooth term *s* with cubic regression splines (*bs* = “cr”) and up to six dimensions (*k* = 6). The performance of both projection methods was evaluated by comparing the RMSEs of the suspect compounds with an *F*-test (*p* < 0.05). This was implemented in R using *var.test()* from *stats* package between LM and GAM. Additionally, the sum of squares of residuals (SS_res_) was calculated as follows (Equation (2)):

(2) $${\mathrm{SS}}_{\mathrm{res}}=\frac{\sum \left({\mathrm{residuals}}^{2}\right)}{n}$$

where *n* is the number of datapoints. The similarities of the projected logRF values were compared with a heatmap with hierarchical clustering of Pearson and Spearman correlation coefficients, implemented using *cor*(method = “pearson” or “spearman”) and *heatmap()* from the *stats* package [46]. The projected logRFs followed a roughly normal distribution based on visual evaluation.

To examine the consistency of projected logRFs across compounds and datasets, an LM was fitted to all compounds (both suspect compounds and calibrants) to anchor the logRFs to the scale of L38. The projected logRF values were examined with respect to their consistency across datasets and compounds, using the SS_data_ or SS_comp_ (Equation (3)) and manual inspection of line plots (tab ‘logRF across datasets’ in dashboard):

(3) $${\mathrm{SS}}_{\mathrm{data}/\mathrm{comp}}=\frac{\sum {\left(\mathrm{logRF}-\mathrm{mean}(\mathrm{logRF})\right)}^{2}}{n}$$

where mean(logRF) is the average projected logRF across the datasets, and *n* is the number of datasets this compound was detected in, or the number of compounds detected in this dataset for analysis across compounds and datasets, respectively.

All correlations were assessed using either Pearson *R*^2^ (where the linear relationship could be assumed, e.g., for the LM projected logRFs) or Spearman *R*^2^ (where the linear relationship could not be assumed), implemented using *cor_test*(method = “pearson” or “spearman”) or *cor*(method = “pearson” or “spearman”) from *rstatix* [39] and *stats* [46] packages, respectively. The code for logRF projection, SS calculations and logRF clustering analysis is available in Code S3–S5.

### 3.5. Dashboard Development

The interactive dashboard was developed in R using *shiny* (v. 1.8.1.1) [48] and *bslib* (v. 0.7.0) [49] packages. In addition, the packages *ggplot2* (v. 3.5.2) [50], *plotly* (v. 4.10.4) [51], *scales* (v. 1.3.0) [52], and *tidyverse* (v. 2.0.0) [53] were used to make the graphs and handle the data. The dashboard is built so that the user can explore the data interactively and look at the prediction errors for specific samples, quantification approaches, datasets, organic modifiers, mobile phase additives, injection volumes, gradient lengths, mass analyzers, spray voltages, and compounds. Moreover, the user can see how the logRFs change depending on the projection method (GAM, LM, raw) and the consistency across datasets. The data are also available in tabulated form, with the possibility to download the curated dataset.

ChatGPT-4o and 5 were consulted during the development of the dashboard. Specifically, ChatGPT-4o was used to provide suggestions on how to implement reactive objects into the dashboard, and ChatGPT-5 was used to provide suggestions on how to implement the statistical tests. All suggestions regarding dashboard functionalities were manually reviewed and tested; suggestions regarding statistical tests were evaluated by the authors.

## 4. Conclusions

The data from an interlaboratory comparison of quantitative LC/ESI/HRMS NTS were comprehensively analyzed to determine: (1) if any of the reported, common LC/ESI/HRMS parameters systematically influenced the observed prediction error; and (2) how the logRFs from different instruments and methods compared to each other. The in-depth analysis with respect to the prediction error of concentration showed that there was no statistical difference between any of the investigated parameters or settings and the fold errors, with two exceptions. Namely, for the parent–TP approach in the low concentrated sample, the use of acid as a mobile phase additive influenced the prediction errors, as did the spray voltage in the high concentrated sample. The comparison of the prediction errors and the number of detected compounds for analysis on the same instrument revealed that using MeOH as an organic modifier possibly resulted in the higher detection frequency of the compounds used in this study compared to the use of MeCN. However, it was impossible to assess whether the difference in detection frequency was mainly a result of the choice of organic modifier, the additive type, the reported pH, or a combination of all.

From the response factor analysis, we could firstly observe that the logRFs became more comparable across a wide range of analytical instruments and methods once they were projected to the same scale using a linear model. Moreover, a weak negative correlation was observed between the Pearson *R*^2^ of the linear fit of projected and anchoring logRFs and the compression of the logRF range. This indicates that the projected logRF range will become narrower as the agreement of the experimental logRFs and the logRF of the anchoring dataset decreases. From the clustering of projected logRFs, one dataset, originating from the only laboratory utilizing nanospray, was noticeably different from the others. Despite discussions about an increased response or similar response for all analytes with nanospray, our results are in contradiction to those claims; the logRF range for this dataset ranged around four orders of magnitude before projection, and two orders of magnitude after projection. Finally, the consistency of the projected logRFs across datasets and compounds were assessed. A weak negative correlation between the SS_comp_ and the projected logRFs indicated that compounds with a lower logRF will have higher variability across different instrumentations.

Due to the interlaboratory setting from which the data presented here were obtained, many parameters co-varied, influencing both the prediction errors and logRFs. This made it difficult to draw any strict conclusions and is one of the main limitations of the study. For improved power in the conclusions, changes in, for example, mobile phase composition and instrumental parameters, and their effect on prediction error and logRF, would need to be investigated systematically.

## Figures and Tables

**Table 1:** The raw and projected logRF ranges for the four datasets with the most narrow projected logRF range.

Dataset	Raw logRF Ranges		Projected logRF Ranges	
	Orders	Min–Max	Orders	Min–Max
L18	2.9	9.7–12.6	1.7	13.2–14.9
L33	3.8	12.2–16.0	1.9	12.3–14.2
L28	3.4	12.4–15.8	2.1	12.9–15.0
L9	3.3	10.5–13.8	2.1	12.7–14.9

**Table 2:** The three datasets with the highest and lowest SS data, and the injection volumes used in the corresponding methods.

Dataset	*n* Compounds Detected	SS_data_	Injection Volume (µL)
L33	42	0.378	1
L38	68	0.267	10
L1	61	0.245	5
L29	69	0.085	10
L12	49	0.085	10
L18	37	0.083	2

**Table 3:** The four compounds with the highest and lowest SS_comp_. Four were chosen, as the compound with the highest and the lowest SS_comp_ was only detected in two datasets; thus, the SS_comp_ may be biased.

Compounds	Type of Compound	Detected in *n* Datasets ^a^	SS_comp_	Average Projected logRF
Sucralose [M+H]^+^	Calibrant	2 ^b^	0.814	11.5
Saccharin	Calibrant	6 ^b^	0.521	11.9
Methomyl	Suspect	20	0.410	13.3
Octocrylene	Calibrant	23	0.383	13.1
Caffeine	Calibrant	35	0.029	13.7
Adenosine	Suspect	25	0.026	13.7
Avermectin B1a [M+H]^+^	Calibrant	4 ^b^	0.010	13.3
Reserpine	Suspect	2 ^b^	0.004	14.1

^a^ Detected refers to detected and left after quality assessment of peaks. ^b^ The compound was detected in very few datasets, which may bias the SS calculation.

## Data Availability

The dashboard is available at https://norman-data.eu/ILS-dashboard. The raw data are available from NORMAN Digital Freezing Platform, refs. [54,55]; the curated dataset with reprocessed peak areas can be downloaded from the dashboard.

## Supplementary Materials

The following supporting information can be downloaded at: https://www.mdpi.com/article/10.3390/molecules31050875/s1.

## Abbreviations

The following abbreviations are used in this manuscript:

RF	Response Factor
LC/HRMS	Liquid Chromatography High Resolution Mass Spectrometry
ESI	Electrospray Ionization
NTS	Non-Target Screening
ML	Machine Learning
*IE*	Ionization Efficiency
MeOH	Methanol
MeCN	Acetonitrile
TP	Transformation Product
RandFor	Random Forest
MLR	Multiple Linear Regression
DDA	Data Dependent Acquisition
MS^1^	Full-Scan Acquisition
LM	Linear Model
GAM	Generalized Additive Model
RMSE	Root Mean Squared Error
SS	Sum of Squares
*R*^2^	Squared correlation coefficient
ToF	Time-of-Flight
RTO	Retention Time Order
RT	Retention Time
