# Supplementary material for: In-Depth Analysis of the Data from an Interlaboratory Study of Quantitative Non-Target Screening—How Do the Instrumental Methods Compare?
Source: Molecules. 2026 Mar 6;31(5):875. doi: 10.3390/molecules31050875 (PMC12986245; doi:10.3390/molecules31050875)
Supplement: Supplementary file 1 [file molecules-31-00875-s001.zip › Code S1_statistical_tests.html]

Statistical tests and pair-wise comparison of datasets


# Statistical tests and pair-wise comparison of datasets

#### Louise Malm

## Read in the libraries

```
library(tidyverse)
library(rstatix)
library(PMCMRplus)
library(scales)
source("theme.R")
```

## Functions

```
scientific_10_text <- function(x) {
  formatted <- gsub("e", "×10^", scales::scientific_format()(x))
  formatted <- gsub("\\^\\+0?", "^", formatted)
  gsub("\\^(-?\\d+)", "<sup>\\1</sup>", formatted)
}
```

## Read in the data

```
results = read_delim("results_all_reprocessed.csv",
                  delim = ",",
                  col_names = T) %>% 
  mutate(Approach = factor(Approach,
                           levels = c("Parent-TP", "Structurally similar",
                                      "Close eluting", "RandFor-IE", "MLR-IE")))

results_2 = read_delim("results_all_reprocessed_clean_colnames.csv",
                     delim = ",",
                     col_names = T)
```

## Wilcoxon rank sum test

### Analyzer type: Orbitrap vs ToF

```
# high concentration
results_orb_tof_h = results_2 %>% 
  filter(sample == "HPLC water high conc.") %>% 
  group_by(approach) %>% 
  wilcox_test(fold_error ~ analyzer_type) %>% 
  adjust_pvalue(method = "bonferroni") %>% 
  add_significance(p.col = "p.adj") %>% 
  select(approach, group1, group2, n1, n2, p, p.adj, p.adj.signif) %>% 
  arrange(approach)

print(results_orb_tof_h)
```

```
## # A tibble: 5 × 8
##   approach             group1   group2    n1    n2     p p.adj p.adj.signif
##   <chr>                <chr>    <chr>  <int> <int> <dbl> <dbl> <chr>       
## 1 Close eluting        Orbitrap ToF      587   424 0.659     1 ns          
## 2 MLR-IE               Orbitrap ToF      527   384 0.404     1 ns          
## 3 Parent-TP            Orbitrap ToF      318   234 0.254     1 ns          
## 4 RandFor-IE           Orbitrap ToF      578   386 0.588     1 ns          
## 5 Structurally similar Orbitrap ToF      587   424 0.605     1 ns
```

```
# effect size and magnitude
eff_size_orb_tof_h = results_2 %>% 
  filter(sample == "HPLC water high conc.") %>% 
  group_by(approach) %>% 
  wilcox_effsize(fold_error ~ analyzer_type)

print(eff_size_orb_tof_h)
```

```
## # A tibble: 5 × 8
##   .y.        group1   group2 effsize approach                n1    n2 magnitude
## * <chr>      <chr>    <chr>    <dbl> <chr>                <int> <int> <ord>    
## 1 fold_error Orbitrap ToF     0.0139 Close eluting          587   424 small    
## 2 fold_error Orbitrap ToF     0.0277 MLR-IE                 527   384 small    
## 3 fold_error Orbitrap ToF     0.0486 Parent-TP              318   234 small    
## 4 fold_error Orbitrap ToF     0.0175 RandFor-IE             578   386 small    
## 5 fold_error Orbitrap ToF     0.0163 Structurally similar   587   424 small
```

```
# low concentration
results_orb_tof_l = results_2 %>% 
  filter(sample == "HPLC water low conc.") %>% 
  group_by(approach) %>% 
  wilcox_test(fold_error ~ analyzer_type) %>% 
  adjust_pvalue(method = "bonferroni") %>% 
  add_significance(p.col = "p.adj") %>% 
  select(approach, group1, group2, n1, n2, p, p.adj, p.adj.signif) %>% 
  arrange(approach)

print(results_orb_tof_l)
```

```
## # A tibble: 5 × 8
##   approach             group1   group2    n1    n2     p p.adj p.adj.signif
##   <chr>                <chr>    <chr>  <int> <int> <dbl> <dbl> <chr>       
## 1 Close eluting        Orbitrap ToF      587   424 0.488 1     ns          
## 2 MLR-IE               Orbitrap ToF      527   384 0.357 1     ns          
## 3 Parent-TP            Orbitrap ToF      318   234 0.2   1     ns          
## 4 RandFor-IE           Orbitrap ToF      578   386 0.025 0.125 ns          
## 5 Structurally similar Orbitrap ToF      587   424 0.684 1     ns
```

```
# effect size and magnitude
eff_size_orb_tof_l = results_2 %>% 
  filter(sample == "HPLC water low conc.") %>% 
  group_by(approach) %>% 
  wilcox_effsize(fold_error ~ analyzer_type)

print(eff_size_orb_tof_l)
```

```
## # A tibble: 5 × 8
##   .y.        group1   group2 effsize approach                n1    n2 magnitude
## * <chr>      <chr>    <chr>    <dbl> <chr>                <int> <int> <ord>    
## 1 fold_error Orbitrap ToF     0.0218 Close eluting          587   424 small    
## 2 fold_error Orbitrap ToF     0.0305 MLR-IE                 527   384 small    
## 3 fold_error Orbitrap ToF     0.0546 Parent-TP              318   234 small    
## 4 fold_error Orbitrap ToF     0.0722 RandFor-IE             578   386 small    
## 5 fold_error Orbitrap ToF     0.0128 Structurally similar   587   424 small
```

### Mobile phase additive: acid vs buffer

```
# high concentration
results_acid_buff_h = results_2 %>% 
  filter(sample == "HPLC water high conc.") %>% 
  group_by(approach) %>% 
  wilcox_test(fold_error ~ mobile_phase_additive) %>% 
  adjust_pvalue(method = "bonferroni") %>% 
  add_significance(p.col = "p.adj") %>% 
  select(approach, group1, group2, n1, n2, p, p.adj, p.adj.signif) %>% 
  arrange(approach)

print(results_acid_buff_h)
```

```
## # A tibble: 5 × 8
##   approach             group1 group2         n1    n2      p p.adj p.adj.signif
##   <chr>                <chr>  <chr>       <int> <int>  <dbl> <dbl> <chr>       
## 1 Close eluting        Acid   Buffer/salt   746   265 0.698  1     ns          
## 2 MLR-IE               Acid   Buffer/salt   676   235 0.349  1     ns          
## 3 Parent-TP            Acid   Buffer/salt   412   140 0.0263 0.132 ns          
## 4 RandFor-IE           Acid   Buffer/salt   737   227 0.0714 0.357 ns          
## 5 Structurally similar Acid   Buffer/salt   746   265 0.208  1     ns
```

```
# effect size and magnitude
eff_size_acid_buff_h = results_2 %>% 
  filter(sample == "HPLC water high conc.") %>% 
  group_by(approach) %>% 
  wilcox_effsize(fold_error ~ mobile_phase_additive)

print(eff_size_acid_buff_h)
```

```
## # A tibble: 5 × 8
##   .y.        group1 group2      effsize approach              n1    n2 magnitude
## * <chr>      <chr>  <chr>         <dbl> <chr>              <int> <int> <ord>    
## 1 fold_error Acid   Buffer/salt  0.0122 Close eluting        746   265 small    
## 2 fold_error Acid   Buffer/salt  0.0311 MLR-IE               676   235 small    
## 3 fold_error Acid   Buffer/salt  0.0946 Parent-TP            412   140 small    
## 4 fold_error Acid   Buffer/salt  0.0581 RandFor-IE           737   227 small    
## 5 fold_error Acid   Buffer/salt  0.0396 Structurally simi…   746   265 small
```

```
# low concentration
results_acid_buff_l = results_2 %>% 
  filter(sample == "HPLC water low conc.") %>% 
  group_by(approach) %>% 
  wilcox_test(fold_error ~ mobile_phase_additive) %>% 
  adjust_pvalue(method = "bonferroni") %>% 
  add_significance(p.col = "p.adj") %>% 
  select(approach, group1, group2, n1, n2, p, p.adj, p.adj.signif) %>% 
  arrange(approach)

print(results_acid_buff_l)
```

```
## # A tibble: 5 × 8
##   approach             group1 group2        n1    n2       p  p.adj p.adj.signif
##   <chr>                <chr>  <chr>      <int> <int>   <dbl>  <dbl> <chr>       
## 1 Close eluting        Acid   Buffer/sa…   746   265 0.622   1      ns          
## 2 MLR-IE               Acid   Buffer/sa…   676   235 0.654   1      ns          
## 3 Parent-TP            Acid   Buffer/sa…   412   140 0.00948 0.0474 *           
## 4 RandFor-IE           Acid   Buffer/sa…   737   227 0.2     1      ns          
## 5 Structurally similar Acid   Buffer/sa…   746   265 0.112   0.56   ns
```

```
# effect size and magnitude
eff_size_acid_buff_l = results_2 %>% 
  filter(sample == "HPLC water low conc.") %>% 
  group_by(approach) %>% 
  wilcox_effsize(fold_error ~ mobile_phase_additive)

print(eff_size_acid_buff_l)
```

```
## # A tibble: 5 × 8
##   .y.        group1 group2      effsize approach              n1    n2 magnitude
## * <chr>      <chr>  <chr>         <dbl> <chr>              <int> <int> <ord>    
## 1 fold_error Acid   Buffer/salt  0.0155 Close eluting        746   265 small    
## 2 fold_error Acid   Buffer/salt  0.0148 MLR-IE               676   235 small    
## 3 fold_error Acid   Buffer/salt  0.110  Parent-TP            412   140 small    
## 4 fold_error Acid   Buffer/salt  0.0413 RandFor-IE           737   227 small    
## 5 fold_error Acid   Buffer/salt  0.0500 Structurally simi…   746   265 small
```

### Organic modifier: MeCN vs MeOH

```
# high concentration
results_MeCN_MeOH_h = results_2 %>% 
  filter(sample == "HPLC water high conc.") %>% 
  group_by(approach) %>% 
  wilcox_test(fold_error ~ org_phase) %>% 
  adjust_pvalue(method = "bonferroni") %>% 
  add_significance(p.col = "p.adj") %>% 
  select(approach, group1, group2, n1, n2, p, p.adj, p.adj.signif) %>% 
  arrange(approach)

print(results_MeCN_MeOH_h)
```

```
## # A tibble: 5 × 8
##   approach             group1 group2    n1    n2      p p.adj p.adj.signif
##   <chr>                <chr>  <chr>  <int> <int>  <dbl> <dbl> <chr>       
## 1 Close eluting        MeCN   MeOH     485   526 0.419  1     ns          
## 2 MLR-IE               MeCN   MeOH     475   436 0.54   1     ns          
## 3 Parent-TP            MeCN   MeOH     273   279 0.582  1     ns          
## 4 RandFor-IE           MeCN   MeOH     483   481 0.0497 0.248 ns          
## 5 Structurally similar MeCN   MeOH     485   526 0.569  1     ns
```

```
# effect size and magnitude
eff_size_MeCN_MeOH_h = results_2 %>% 
  filter(sample == "HPLC water high conc.") %>% 
  group_by(approach) %>% 
  wilcox_effsize(fold_error ~ org_phase)

print(eff_size_MeCN_MeOH_h)
```

```
## # A tibble: 5 × 8
##   .y.        group1 group2 effsize approach                n1    n2 magnitude
## * <chr>      <chr>  <chr>    <dbl> <chr>                <int> <int> <ord>    
## 1 fold_error MeCN   MeOH    0.0254 Close eluting          485   526 small    
## 2 fold_error MeCN   MeOH    0.0203 MLR-IE                 475   436 small    
## 3 fold_error MeCN   MeOH    0.0235 Parent-TP              273   279 small    
## 4 fold_error MeCN   MeOH    0.0632 RandFor-IE             483   481 small    
## 5 fold_error MeCN   MeOH    0.0179 Structurally similar   485   526 small
```

```
# low concentration
results_MeCN_MeOH_l = results_2 %>% 
  filter(sample == "HPLC water low conc.") %>% 
  group_by(approach) %>% 
  wilcox_test(fold_error ~ org_phase) %>% 
  adjust_pvalue(method = "bonferroni") %>% 
  add_significance(p.col = "p.adj") %>% 
  select(approach, group1, group2, n1, n2, p, p.adj, p.adj.signif) %>% 
  arrange(approach)

print(results_MeCN_MeOH_l)
```

```
## # A tibble: 5 × 8
##   approach             group1 group2    n1    n2     p p.adj p.adj.signif
##   <chr>                <chr>  <chr>  <int> <int> <dbl> <dbl> <chr>       
## 1 Close eluting        MeCN   MeOH     485   526 0.809     1 ns          
## 2 MLR-IE               MeCN   MeOH     475   436 0.599     1 ns          
## 3 Parent-TP            MeCN   MeOH     273   279 0.792     1 ns          
## 4 RandFor-IE           MeCN   MeOH     483   481 0.589     1 ns          
## 5 Structurally similar MeCN   MeOH     485   526 0.84      1 ns
```

```
# effect size and magnitude
eff_size_MeCN_MeOH_l = results_2 %>% 
  filter(sample == "HPLC water low conc.") %>% 
  group_by(approach) %>% 
  wilcox_effsize(fold_error ~ org_phase)

print(eff_size_MeCN_MeOH_l)
```

```
## # A tibble: 5 × 8
##   .y.        group1 group2 effsize approach                n1    n2 magnitude
## * <chr>      <chr>  <chr>    <dbl> <chr>                <int> <int> <ord>    
## 1 fold_error MeCN   MeOH   0.00759 Close eluting          485   526 small    
## 2 fold_error MeCN   MeOH   0.0174  MLR-IE                 475   436 small    
## 3 fold_error MeCN   MeOH   0.0112  Parent-TP              273   279 small    
## 4 fold_error MeCN   MeOH   0.0174  RandFor-IE             483   481 small    
## 5 fold_error MeCN   MeOH   0.00634 Structurally similar   485   526 small
```

### Gradient length: <=25 min, >25 min

```
# high concentration
results_grad_length_h = results_2 %>% 
  filter(sample == "HPLC water high conc.") %>% 
  group_by(approach) %>% 
  wilcox_test(fold_error ~ grad_length_min) %>% 
  adjust_pvalue(method = "bonferroni") %>% 
  add_significance(p.col = "p.adj") %>% 
  select(approach, group1, group2, n1, n2, p, p.adj, p.adj.signif) %>% 
  arrange(approach)

print(results_grad_length_h)
```

```
## # A tibble: 5 × 8
##   approach             group1 group2    n1    n2     p p.adj p.adj.signif
##   <chr>                <chr>  <chr>  <int> <int> <dbl> <dbl> <chr>       
## 1 Close eluting        ?25    >25      503   508 0.545   1   ns          
## 2 MLR-IE               ?25    >25      432   479 0.14    0.7 ns          
## 3 Parent-TP            ?25    >25      282   270 0.999   1   ns          
## 4 RandFor-IE           ?25    >25      463   501 0.12    0.6 ns          
## 5 Structurally similar ?25    >25      503   508 0.713   1   ns
```

```
# effect size and magnitude
eff_size_grad_length_h = results_2 %>% 
  filter(sample == "HPLC water high conc.") %>% 
  group_by(approach) %>% 
  wilcox_effsize(fold_error ~ grad_length_min)

print(eff_size_grad_length_h)
```

```
## # A tibble: 5 × 8
##   .y.        group1 group2   effsize approach                n1    n2 magnitude
## * <chr>      <chr>  <chr>      <dbl> <chr>                <int> <int> <ord>    
## 1 fold_error ?25    >25    0.0190    Close eluting          503   508 small    
## 2 fold_error ?25    >25    0.0490    MLR-IE                 432   479 small    
## 3 fold_error ?25    >25    0.0000454 Parent-TP              282   270 small    
## 4 fold_error ?25    >25    0.0501    RandFor-IE             463   501 small    
## 5 fold_error ?25    >25    0.0116    Structurally similar   503   508 small
```

```
# low concentration
results_grad_length_l = results_2 %>% 
  filter(sample == "HPLC water low conc.") %>% 
  group_by(approach) %>% 
  wilcox_test(fold_error ~ grad_length_min) %>% 
  adjust_pvalue(method = "bonferroni") %>% 
  add_significance(p.col = "p.adj") %>% 
  select(approach, group1, group2, n1, n2, p, p.adj, p.adj.signif) %>% 
  arrange(approach)

print(results_grad_length_l)
```

```
## # A tibble: 5 × 8
##   approach             group1 group2    n1    n2      p  p.adj p.adj.signif
##   <chr>                <chr>  <chr>  <int> <int>  <dbl>  <dbl> <chr>       
## 1 Close eluting        ?25    >25      503   508 0.202  1      ns          
## 2 MLR-IE               ?25    >25      432   479 0.0113 0.0565 ns          
## 3 Parent-TP            ?25    >25      282   270 0.989  1      ns          
## 4 RandFor-IE           ?25    >25      463   501 0.0567 0.284  ns          
## 5 Structurally similar ?25    >25      503   508 0.703  1      ns
```

```
# effect size and magnitude
eff_size_grad_length_l = results_2 %>% 
  filter(sample == "HPLC water low conc.") %>% 
  group_by(approach) %>% 
  wilcox_effsize(fold_error ~ grad_length_min)

print(eff_size_grad_length_l)
```

```
## # A tibble: 5 × 8
##   .y.        group1 group2  effsize approach                n1    n2 magnitude
## * <chr>      <chr>  <chr>     <dbl> <chr>                <int> <int> <ord>    
## 1 fold_error ?25    >25    0.0402   Close eluting          503   508 small    
## 2 fold_error ?25    >25    0.0840   MLR-IE                 432   479 small    
## 3 fold_error ?25    >25    0.000614 Parent-TP              282   270 small    
## 4 fold_error ?25    >25    0.0614   RandFor-IE             463   501 small    
## 5 fold_error ?25    >25    0.0120   Structurally similar   503   508 small
```

## Friedman test

### Injection volume: <10 uL, 10-25 uL, >25 uL

#### Is there a difference in the mean error across the injection volumes?

H0: No significant difference in the mean error for the different
injection volumes

Preparing the data

```
inj_vol_dataset_filtered = results_2 %>%
  filter(!dataset %in% c("L12", "L20", "L35", "L37")) %>%  # filtered since there were no results for MLR_IE approach for these datasets
  group_by(approach, sample, inj_vol_uL) %>%
  mutate(median_fold_error = median(fold_error)) %>%
  ungroup() %>% 
  select(approach, median_fold_error, sample, inj_vol_uL)

inj_vol_h = inj_vol_dataset_filtered %>% 
  filter(sample == "HPLC water high conc.")

inj_vol_h = inj_vol_h[!duplicated(inj_vol_h), ]

inj_vol_l = inj_vol_dataset_filtered %>% 
  filter(sample == "HPLC water low conc.")

inj_vol_l = inj_vol_l[!duplicated(inj_vol_l), ]
```

High concentration spike in HPLC water

```
# calculating sample sizes
sample_size_inj_vol_h <- results_2 %>%
  filter(!dataset %in% c("L12", "L20", "L35", "L37")) %>%
  filter(sample == "HPLC water high conc.") %>%
  group_by(inj_vol_uL) %>%
  summarise(
    n_observations = n(),
    n_datasets = n_distinct(dataset),
    .groups = 'drop'
  )

print(sample_size_inj_vol_h)
```

```
## # A tibble: 3 × 3
##   inj_vol_uL n_observations n_datasets
##   <chr>               <int>      <int>
## 1 10-25                1773         14
## 2 <10                  1332         11
## 3 >25                   990          8
```

```
friedman_sample_size_inj_vol_h <- results_2 %>%
  filter(!dataset %in% c("L12", "L20", "L35", "L37")) %>%
  filter(sample == "HPLC water high conc.") %>%
  summarise(
    n_blocks = n_distinct(approach),           # Number of blocks (approaches)
    n_treatments = n_distinct(inj_vol_uL),     # Number of treatments (injection volumes)
    n_per_block_treatment = n() / (n_distinct(approach) * n_distinct(inj_vol_uL))  # Observations per cell
  )

print(friedman_sample_size_inj_vol_h)
```

```
## # A tibble: 1 × 3
##   n_blocks n_treatments n_per_block_treatment
##      <int>        <int>                 <dbl>
## 1        5            3                   273
```

```
# Friedman test
friedmanTest(y = inj_vol_h$median_fold_error,
             groups = inj_vol_h$inj_vol_uL,
             blocks = inj_vol_h$approach)
```

```
## 
##  Friedman rank sum test
## 
## data:  y, groups and blocks
## Friedman chi-squared = 1.6, df = 2, p-value = 0.4493
```

*p* > 0.05 –> no statistical difference

Low concentration spike in HPLC water

```
# calculating sample sizes
sample_size_inj_vol_l <- results_2 %>%
  filter(!dataset %in% c("L12", "L20", "L35", "L37")) %>%
  filter(sample == "HPLC water low conc.") %>%
  group_by(inj_vol_uL) %>%
  summarise(
    n_observations = n(),
    n_datasets = n_distinct(dataset),
    .groups = 'drop'
  )

print(sample_size_inj_vol_l)
```

```
## # A tibble: 3 × 3
##   inj_vol_uL n_observations n_datasets
##   <chr>               <int>      <int>
## 1 10-25                1773         14
## 2 <10                  1332         11
## 3 >25                   990          8
```

```
friedman_sample_size_inj_vol_l <- results_2 %>%
  filter(!dataset %in% c("L12", "L20", "L35", "L37")) %>%
  filter(sample == "HPLC water low conc.") %>%
  summarise(
    n_blocks = n_distinct(approach),           # Number of blocks (approaches)
    n_treatments = n_distinct(inj_vol_uL),     # Number of treatments (injection volumes)
    n_per_block_treatment = n() / (n_distinct(approach) * n_distinct(inj_vol_uL))  # Observations per cell
  )

print(friedman_sample_size_inj_vol_l)
```

```
## # A tibble: 1 × 3
##   n_blocks n_treatments n_per_block_treatment
##      <int>        <int>                 <dbl>
## 1        5            3                   273
```

```
# Friedman test
friedmanTest(y = inj_vol_l$median_fold_error,
             groups = inj_vol_l$inj_vol_uL,
             blocks = inj_vol_l$approach)
```

```
## 
##  Friedman rank sum test
## 
## data:  y, groups and blocks
## Friedman chi-squared = 3.6, df = 2, p-value = 0.1653
```

*p* > 0.05 –> no statistical difference

### Spray voltage: <3.0 kV, 3.0-3.5 kV, > 3.5 kV

#### Is there a difference in the mean error across the spray voltages?

H0: No statistical difference in the mean error for the different
spray voltages

Preparing the data

```
spray_volt_dataset_filtered = results_2 %>%
  filter(!dataset %in% c("L12", "L20", "L35", "L37")) %>%  # filtered since there were no results for MLR_IE approach for these labs
  group_by(approach, sample, spray_volt_kV) %>%
  mutate(median_fold_error = median(fold_error)) %>%
  ungroup() %>% 
  select(approach, median_fold_error, sample, spray_volt_kV)

spray_volt_h = spray_volt_dataset_filtered %>% 
  filter(sample == "HPLC water high conc.")

spray_volt_h = spray_volt_h[!duplicated(spray_volt_h), ]

spray_volt_l = spray_volt_dataset_filtered %>% 
  filter(sample == "HPLC water low conc.")

spray_volt_l = spray_volt_l[!duplicated(spray_volt_l), ]
```

High concentration spike in HPLC water

```
# calculating sample sizes
sample_size_spray_volt_h <- results_2 %>%
  filter(!dataset %in% c("L12", "L20", "L35", "L37")) %>%
  filter(sample == "HPLC water high conc.") %>%
  group_by(spray_volt_kV) %>%
  summarise(
    n_observations = n(),
    n_datasets = n_distinct(dataset),
    .groups = 'drop'
  )

print(sample_size_spray_volt_h)
```

```
## # A tibble: 3 × 3
##   spray_volt_kV n_observations n_datasets
##   <chr>                  <int>      <int>
## 1 3.0-3.5                 2341         18
## 2 <3.0                     578          5
## 3 >3.5                    1176         10
```

```
friedman_sample_size_spray_volt_h <- results_2 %>%
  filter(!dataset %in% c("L12", "L20", "L35", "L37")) %>%
  filter(sample == "HPLC water high conc.") %>%
  summarise(
    n_blocks = n_distinct(approach),           # Number of blocks (approaches)
    n_treatments = n_distinct(spray_volt_kV),     # Number of treatments (injection volumes)
    n_per_block_treatment = n() / (n_distinct(approach) * n_distinct(spray_volt_kV))  # Observations per cell
  )

print(friedman_sample_size_spray_volt_h)
```

```
## # A tibble: 1 × 3
##   n_blocks n_treatments n_per_block_treatment
##      <int>        <int>                 <dbl>
## 1        5            3                   273
```

```
# Friedman test
friedmanTest(y = spray_volt_h$median_fold_error,
             groups = spray_volt_h$spray_volt_kV,
             blocks = spray_volt_h$approach)
```

```
## 
##  Friedman rank sum test
## 
## data:  y, groups and blocks
## Friedman chi-squared = 7.6, df = 2, p-value = 0.02237
```

*p* < 0.05 –> there is a statistical difference

Nemenyi’s posthoc test

```
# Nemenyi test
spray_volt_h_nem_res = frdAllPairsNemenyiTest(y = spray_volt_h$median_fold_error,
                                           groups = spray_volt_h$spray_volt_kV,
                                           blocks = spray_volt_h$approach)

spray_volt_h_nem_res$p.value
```

```
##               <3.0      >3.5
## >3.5    0.94637039        NA
## 3.0-3.5 0.03066275 0.0688869
```

–> There is a statistical difference in the error for labs using a
spray voltage lower than 3.0 kV compared to labs using spray voltages
between 3.0 and 3.5 kV (p < 0.05).

Low concentration spike in HPLC water

```
# calculating sample sizes
sample_size_spray_volt_l <- results_2 %>%
  filter(!dataset %in% c("L12", "L20", "L35", "L37")) %>%
  filter(sample == "HPLC water high conc.") %>%
  group_by(spray_volt_kV) %>%
  summarise(
    n_observations = n(),
    n_datasets = n_distinct(dataset),
    .groups = 'drop'
  )

print(sample_size_spray_volt_l)
```

```
## # A tibble: 3 × 3
##   spray_volt_kV n_observations n_datasets
##   <chr>                  <int>      <int>
## 1 3.0-3.5                 2341         18
## 2 <3.0                     578          5
## 3 >3.5                    1176         10
```

```
friedman_sample_size_spray_volt_l <- results_2 %>%
  filter(!dataset %in% c("L12", "L20", "L35", "L37")) %>%
  filter(sample == "HPLC water high conc.") %>%
  summarise(
    n_blocks = n_distinct(approach),           # Number of blocks (approaches)
    n_treatments = n_distinct(spray_volt_kV),     # Number of treatments (injection volumes)
    n_per_block_treatment = n() / (n_distinct(approach) * n_distinct(spray_volt_kV))  # Observations per cell
  )

print(friedman_sample_size_spray_volt_l)
```

```
## # A tibble: 1 × 3
##   n_blocks n_treatments n_per_block_treatment
##      <int>        <int>                 <dbl>
## 1        5            3                   273
```

```
# Friedman test
friedmanTest(y = spray_volt_l$median_fold_error,
             groups = spray_volt_l$spray_volt_kV,
             blocks = spray_volt_l$approach)
```

```
## 
##  Friedman rank sum test
## 
## data:  y, groups and blocks
## Friedman chi-squared = 2.8, df = 2, p-value = 0.2466
```

p > 0.05 –> no statistical difference

## Error statistics and analysis same instrument

### MeOH vs MeCN as organic modifier

```
L10_L14_data <- results %>% 
  filter(Dataset %in% c("L10", "L14"))

error_stat_L10_L14 = L10_L14_data %>%
  group_by(Approach, Dataset) %>%
  summarise(
    mean_error = mean(`Fold error`),
    median_error = median(`Fold error`),
    max_error = max(`Fold error`),
    quantile_error = quantile(`Fold error`, probs = c(0.95)),
    n_dp = length(`Fold error`),
    n_dp_less_than_ten = length(`Fold error`[`Fold error`<10 & `Fold error`>1]),
    percentage_less_than_ten = (length(`Fold error`[`Fold error`<10 & `Fold error`>1]))/(length(`Fold error`)))%>%
  ungroup()
```

Visualization with boxplots

```
L10_L14 <- ggplot(L10_L14_data %>% 
         filter(Sample == "HPLC water high conc.")) +
  geom_rect(mapping = aes(ymin = 0,
                          ymax = 10^1,
                          xmin = -Inf,
                          xmax = Inf),
            fill = "#C1DBB3") +
  geom_boxplot(mapping = aes(y = `Fold error`,
                             x = Approach,
                             fill = Dataset),
               alpha = 0.8,
               color = "#262626",
               outlier.color = "#262626",
               outlier.alpha = 0.7) +
  scale_fill_manual(values = c("#8C2155", "#F7DEEA")) +
  scale_x_discrete(labels=c("Structurally similar" = "Structurally similar",
                            "Parent-TP" = "Parent-TP",
                            "Close eluting" = "Close eluting",
                            "RandFor-IE" = substitute(paste("RandFor-", italic("IE "))),
                            "MLR-IE" = substitute(paste("MLR-", italic("IE "))))) +
  scale_y_log10(labels = trans_format("log10",
                                      math_format(10^.x)),
                breaks = c(10^1, 10^2, 10^3),
                limits = c(1, 1*10^3)) +
  labs(y = "Fold error",
       x = "") +
  my_theme +
  theme(aspect.ratio = 0.35,
        legend.position = "none",
        axis.title = element_text(family = font,
                                 size = 12,
                                 color = basecolor),
        axis.text.x = element_text(angle = 45,
                                   hjust = 1))

L10_L14
```

Distribution of errors

```
ggplot(L10_L14_data %>% 
         filter(Approach == "MLR-IE",
                Sample == "HPLC water high conc."), 
       aes(x = `Fold error`, fill = Dataset)) +
  scale_fill_manual(values = c("#659D8B", "#8C2155")) +
  geom_histogram(alpha = 0.6, position = "identity")
```

The errors are not normally distributed for any approach, we can
assume the same for all datasets. Therefore we will use wilcoxon rank
sum test

```
results_L10_L14_h <- L10_L14_data %>% 
  filter(Sample == "HPLC water high conc.") %>%
  group_by(Approach) %>% 
  wilcox_test(`Fold error` ~ Dataset) %>% 
  adjust_pvalue(method = "bonferroni") %>% 
  add_significance(p.col = "p") %>% 
  select(Approach, group1, group2, n1, n2, p, p.adj, p.signif) %>% 
  arrange(Approach)

print(results_L10_L14_h)
```

```
## # A tibble: 5 × 8
##   Approach             group1 group2    n1    n2     p p.adj p.signif
##   <fct>                <chr>  <chr>  <int> <int> <dbl> <dbl> <chr>   
## 1 Parent-TP            L10    L14       19    14 0.461  1    ns      
## 2 Structurally similar L10    L14       35    28 0.655  1    ns      
## 3 Close eluting        L10    L14       35    28 0.17   0.85 ns      
## 4 RandFor-IE           L10    L14       34    27 0.768  1    ns      
## 5 MLR-IE               L10    L14       35    28 0.706  1    ns
```

```
results_L10_L14_l <- L10_L14_data %>% 
  filter(Sample == "HPLC water low conc.") %>%
  group_by(Approach) %>% 
  wilcox_test(`Fold error` ~ Dataset) %>% 
  adjust_pvalue(method = "bonferroni") %>% 
  add_significance(p.col = "p") %>% 
  select(Approach, group1, group2, n1, n2, p, p.adj, p.signif) %>% 
  arrange(Approach)

print(results_L10_L14_l)
```

```
## # A tibble: 5 × 8
##   Approach             group1 group2    n1    n2     p p.adj p.signif
##   <fct>                <chr>  <chr>  <int> <int> <dbl> <dbl> <chr>   
## 1 Parent-TP            L10    L14       19    14 0.843     1 ns      
## 2 Structurally similar L10    L14       35    28 0.789     1 ns      
## 3 Close eluting        L10    L14       35    28 0.325     1 ns      
## 4 RandFor-IE           L10    L14       34    27 0.629     1 ns      
## 5 MLR-IE               L10    L14       35    28 0.568     1 ns
```

### Data acquired with DDA vs MS1

```
L22_L40_data <- results %>% 
  filter(Dataset %in% c("L22", "L40"))

error_stat_L22_L40 <- L22_L40_data %>% 
  group_by(Approach, Dataset) %>% 
  summarise(
    mean_error = mean(`Fold error`),
    median_error = median(`Fold error`),
    max_error = max(`Fold error`),
    quantile_error = quantile(`Fold error`, probs = c(0.95)),
    n_dp = length(`Fold error`),
    n_dp_less_than_ten = length(`Fold error`[`Fold error`<10 & `Fold error`>1]),
    percentage_less_than_ten = (length(`Fold error`[`Fold error`<10 & `Fold error`>1]))/(length(`Fold error`))) %>% 
  ungroup()
```

Visualization with boxplots

```
L22_L40 <- ggplot(L22_L40_data %>% 
         filter(Sample == "HPLC water high conc.")) +
  geom_rect(mapping = aes(ymin = 0,
                          ymax = 10^1,
                          xmin = -Inf,
                          xmax = Inf),
            fill = "#C1DBB3") +
  geom_boxplot(mapping = aes(y = `Fold error`,
                             x = Approach,
                             fill = Dataset),
               alpha = 0.8,
               color = "#262626",
               outlier.color = "#262626",
               outlier.alpha = 0.7) +
  scale_y_log10(labels = trans_format("log10",
                                      math_format(10^.x)),
                breaks = c(10^1, 10^2, 10^3),
                limits = c(1, 1*10^3)) +
  scale_fill_manual(values = c("#8C2155", "#F7DEEA")) +
  scale_x_discrete(labels=c("Structurally similar" = "Structurally similar",
                            "Parent-TP" = "Parent-TP",
                            "Close eluting" = "Close eluting",
                            "RandFor-IE" = substitute(paste("RandFor-", italic("IE "))),
                            "MLR-IE" = substitute(paste("MLR-", italic("IE "))))) +
  labs(y = "Fold error",
       x = "") +
  my_theme +
  theme(aspect.ratio = 0.35,
        legend.position = "none",
        axis.title = element_text(family = font,
                                  size = 12,
                                  color = basecolor),
        axis.text.x = element_text(angle = 45,
                                   hjust = 1))

L22_L40
```

We can assume non-normal distribution of the errors –> Wilcoxon
rank sum test

```
results_L22_L40_h <- L22_L40_data %>% 
  filter(Sample == "HPLC water high conc.") %>% 
  group_by(Approach) %>% 
  wilcox_test(`Fold error` ~ Dataset) %>% 
  adjust_pvalue(method = "bonferroni") %>% 
  add_significance(p.col = "p") %>% 
  select(Approach, group1, group2, n1, n2, p, p.adj, p.signif) %>% 
  arrange(Approach)

print(results_L22_L40_h)
```

```
## # A tibble: 5 × 8
##   Approach             group1 group2    n1    n2     p p.adj p.signif
##   <fct>                <chr>  <chr>  <int> <int> <dbl> <dbl> <chr>   
## 1 Parent-TP            L22    L40       11    13 0.733     1 ns      
## 2 Structurally similar L22    L40       24    29 0.908     1 ns      
## 3 Close eluting        L22    L40       24    29 0.797     1 ns      
## 4 RandFor-IE           L22    L40       23    28 0.8       1 ns      
## 5 MLR-IE               L22    L40       24    29 0.342     1 ns
```

```
results_L22_L40_l <- L22_L40_data %>% 
  filter(Sample == "HPLC water low conc.") %>% 
  group_by(Approach) %>% 
  wilcox_test(`Fold error` ~ Dataset) %>% 
  adjust_pvalue(method = "bonferroni") %>% 
  add_significance(p.col = "p") %>% 
  select(Approach, group1, group2, n1, n2, p, p.adj, p.signif) %>% 
  arrange(Approach)

print(results_L22_L40_l)
```

```
## # A tibble: 5 × 8
##   Approach             group1 group2    n1    n2     p p.adj p.signif
##   <fct>                <chr>  <chr>  <int> <int> <dbl> <dbl> <chr>   
## 1 Parent-TP            L22    L40       11    13 0.865     1 ns      
## 2 Structurally similar L22    L40       24    29 0.677     1 ns      
## 3 Close eluting        L22    L40       24    29 0.77      1 ns      
## 4 RandFor-IE           L22    L40       23    28 0.673     1 ns      
## 5 MLR-IE               L22    L40       24    29 0.351     1 ns
```
