# Supplementary material for: In-Depth Analysis of the Data from an Interlaboratory Study of Quantitative Non-Target Screening—How Do the Instrumental Methods Compare?
Source: Molecules. 2026 Mar 6;31(5):875. doi: 10.3390/molecules31050875 (PMC12986245; doi:10.3390/molecules31050875)
Supplement: Supplementary file 1 [file molecules-31-00875-s001.zip › Code S2_RandFor_model_error.html]

2\_RandFor\_model\_error


# Random Forest model to predict error¶

### Libraries¶

In [1]:

```
import pandas as pd
import numpy as np
from sklearn.model_selection import GroupShuffleSplit
from sklearn.ensemble import RandomForestRegressor
from sklearn.metrics import mean_squared_error, r2_score, mean_absolute_error
from sklearn.preprocessing import LabelEncoder
import matplotlib.pyplot as plt
import shap
```

### Read in data¶

In [2]:

```
df = pd.read_csv('results_all.csv')

print("Original dataset shape:", df.shape)
```

```
Original dataset shape: (25264, 19)
```

## For log(fold error) predictions¶

#### Filter and log-transform the data¶

In [3]:

```
df_filtered = df[df['sample'] == 'HPLC_high'].copy()

print("Filtered dataset shape:", df_filtered.shape)
```

```
Filtered dataset shape: (4449, 19)
```

In [4]:

```
df_filtered['log_fold_error'] = np.log10(df_filtered['fold_error'])

print("\nlog_fold_error statistics:")
print(df_filtered['log_fold_error'].describe())
```

```
log_fold_error statistics:
count    4449.000000
mean        0.632217
std         0.550014
min         0.000000
25%         0.216861
50%         0.490675
75%         0.899520
max         4.558000
Name: log_fold_error, dtype: float64
```

In [5]:

```
# Select only the columns we need for features and target
feature_columns = ['compound', 'approach', 'analyzer', 'mobile_phase_add', 'org_phase', 
                   'inj_vol_ul', 'spray_volt_kv', 'grad_length_min']
target_column = 'log_fold_error'
```

### Feature matrix¶

In [6]:

```
X = df_filtered[feature_columns].copy()
y_log = df_filtered[target_column].copy()
```

In [7]:

```
# Check for missing values
print("\nMissing values in features:")
print(X.isnull().sum())
print("\nMissing values in target:")
print(y_log.isnull().sum())
```

```
Missing values in features:
compound            0
approach            0
analyzer            0
mobile_phase_add    0
org_phase           0
inj_vol_ul          0
spray_volt_kv       0
grad_length_min     0
dtype: int64

Missing values in target:
0
```

In [8]:

```
# Display unique values for each categorical column
print("\n=== Unique values per feature ===")
for col in feature_columns:
    print(f"\n{col}: {X[col].nunique()} unique values")
    print(X[col].value_counts())
```

```
=== Unique values per feature ===

compound: 41 unique values
compound
2-methylbenzothiazole                       156
Atrazine-2-hydroxy                          156
Metolachlor-ESA                             153
Carbamazepine-10,11-epoxide                 151
Atrazine-desethyl                           150
Simazine-2-hydroxy                          149
5-methyl-1H-benzotriazole                   148
5-chlorobenzotriazole                       148
2-(methylthio)benzothiazole                 147
2-aminobenzothiazole                        146
Theophylline                                145
Atrazine-desisopropyl                       145
2-hydroxybenzothiazole                      144
Monuron                                     139
Metazachlor                                 136
Benzotriazole-5-carboxylic acid             134
Metolachlor-OA                              132
10,11-dihydro-10-hydroxycarbamazepine       126
Climbazole                                  124
Irgarol                                     121
Thiabendazole                               120
Omethoate                                   120
Phenazine                                   117
Methidathion                                116
Atrazine-desethyl-desisopropyl              112
Sudan I                                     109
Sebuthylazine                               109
Atrazine-desethyl-2-hydroxy                 101
Adenosine                                    96
Chlorpyrifos                                 89
Methomyl                                     77
Benzothiazole                                77
Acephate                                     72
Ketoconazole                                 68
Atrazine-desisopropyl-2-hydroxy              59
Naproxen                                     39
Melamine                                     35
Clotrimazole                                 32
Metformin                                    24
Atrazine-desethyl-desisopropyl-2-hydroxy     19
Reserpine                                     8
Name: count, dtype: int64

approach: 5 unique values
approach
close_elut    1011
struct_sim    1011
randfor_IE     964
MLR_IE         911
parent         552
Name: count, dtype: int64

analyzer: 2 unique values
analyzer
orb    2597
tof    1852
Name: count, dtype: int64

mobile_phase_add: 2 unique values
mobile_phase_add
acid      3317
buffer    1132
Name: count, dtype: int64

org_phase: 2 unique values
org_phase
meoh    2248
mecn    2201
Name: count, dtype: int64

inj_vol_ul: 3 unique values
inj_vol_ul
10_ul_below    2651
above_25_ul    1198
10_25_ul        600
Name: count, dtype: int64

spray_volt_kv: 3 unique values
spray_volt_kv
3.0_3.5_kv      2000
above_3.5_kv    1391
3.0_kv_below    1058
Name: count, dtype: int64

grad_length_min: 2 unique values
grad_length_min
above_25_min    2266
25_min_below    2183
Name: count, dtype: int64
```

### Encode categorical variables¶

- For 'compound', we'll use Label Encoding since it's the compound name
- For the other features, we can also use Label Encoding or One-Hot Encoding
- Let's use Label Encoding for all to keep it simple

In [9]:

```
label_encoders = {}
X_encoded = X.copy()

for col in feature_columns:
    le = LabelEncoder()
    X_encoded[col] = le.fit_transform(X[col].astype(str))
    label_encoders[col] = le
    print(f"\nEncoded {col}:")
    print(f"  Original values: {le.classes_}")
    print(f"  Encoded as: {list(range(len(le.classes_)))}")
```

```
Encoded compound:
  Original values: ['10,11-dihydro-10-hydroxycarbamazepine' '2-(methylthio)benzothiazole'
 '2-aminobenzothiazole' '2-hydroxybenzothiazole' '2-methylbenzothiazole'
 '5-chlorobenzotriazole' '5-methyl-1H-benzotriazole' 'Acephate'
 'Adenosine' 'Atrazine-2-hydroxy' 'Atrazine-desethyl'
 'Atrazine-desethyl-2-hydroxy' 'Atrazine-desethyl-desisopropyl'
 'Atrazine-desethyl-desisopropyl-2-hydroxy' 'Atrazine-desisopropyl'
 'Atrazine-desisopropyl-2-hydroxy' 'Benzothiazole'
 'Benzotriazole-5-carboxylic acid' 'Carbamazepine-10,11-epoxide'
 'Chlorpyrifos' 'Climbazole' 'Clotrimazole' 'Irgarol' 'Ketoconazole'
 'Melamine' 'Metazachlor' 'Metformin' 'Methidathion' 'Methomyl'
 'Metolachlor-ESA' 'Metolachlor-OA' 'Monuron' 'Naproxen' 'Omethoate'
 'Phenazine' 'Reserpine' 'Sebuthylazine' 'Simazine-2-hydroxy' 'Sudan I'
 'Theophylline' 'Thiabendazole']
  Encoded as: [0, 1, 2, 3, 4, 5, 6, 7, 8, 9, 10, 11, 12, 13, 14, 15, 16, 17, 18, 19, 20, 21, 22, 23, 24, 25, 26, 27, 28, 29, 30, 31, 32, 33, 34, 35, 36, 37, 38, 39, 40]

Encoded approach:
  Original values: ['MLR_IE' 'close_elut' 'parent' 'randfor_IE' 'struct_sim']
  Encoded as: [0, 1, 2, 3, 4]

Encoded analyzer:
  Original values: ['orb' 'tof']
  Encoded as: [0, 1]

Encoded mobile_phase_add:
  Original values: ['acid' 'buffer']
  Encoded as: [0, 1]

Encoded org_phase:
  Original values: ['mecn' 'meoh']
  Encoded as: [0, 1]

Encoded inj_vol_ul:
  Original values: ['10_25_ul' '10_ul_below' 'above_25_ul']
  Encoded as: [0, 1, 2]

Encoded spray_volt_kv:
  Original values: ['3.0_3.5_kv' '3.0_kv_below' 'above_3.5_kv']
  Encoded as: [0, 1, 2]

Encoded grad_length_min:
  Original values: ['25_min_below' 'above_25_min']
  Encoded as: [0, 1]
```

### Random forest¶

In [10]:

```
# Split the data into training and testing sets
groups = df_filtered['dataset']

gss = GroupShuffleSplit(n_splits=1, test_size=0.2, random_state=42)
train_idx, test_idx = next(gss.split(X_encoded, y_log, groups=groups))

X_train = X_encoded.iloc[train_idx]
X_test = X_encoded.iloc[test_idx]
y_train_log = y_log.iloc[train_idx]
y_test_log = y_log.iloc[test_idx]

print(f"\nTraining set size: {X_train.shape[0]}")
print(f"Test set size: {X_test.shape[0]}")
print(f"\nDatasets in training set: {df_filtered['dataset'].iloc[train_idx].unique()}")
print(f"Datasets in test set: {df_filtered['dataset'].iloc[test_idx].unique()}")
```

```
Training set size: 3407
Test set size: 1042

Datasets in training set: ['L1' 'L11' 'L12' 'L15' 'L17' 'L19' 'L21' 'L22' 'L25' 'L29' 'L3' 'L32'
 'L33' 'L34' 'L37' 'L38' 'L40' 'L41' 'L6' 'L7' 'L10' 'L18' 'L24' 'L26'
 'L5' 'L9' 'L28' 'L20' 'L31']
Datasets in test set: ['L16' 'L23' 'L27' 'L35' 'L36' 'L8' 'L14' 'L39']
```

In [11]:

```
# Initialize and train the Random Forest model
rf_model = RandomForestRegressor(
    n_estimators=100,
    max_depth=None,
    min_samples_split=2,
    min_samples_leaf=1,
    random_state=42,
    n_jobs=-1
)
```

In [12]:

```
# Train the model
print("\nTraining the model...")
rf_model.fit(X_train, y_train_log)
```

```
Training the model...
```

Out[12]:

```
RandomForestRegressor(n_jobs=-1, random_state=42)
```

**In a Jupyter environment, please rerun this cell to show the HTML representation or trust the notebook.   
On GitHub, the HTML representation is unable to render, please try loading this page with nbviewer.org.**

RandomForestRegressor

?Documentation for RandomForestRegressoriFitted

Parameters

|  |  |  |
| --- | --- | --- |
|  | n\_estimators n\_estimators: int, default=100  The number of trees in the forest.  .. versionchanged:: 0.22  The default value of ``n\_estimators`` changed from 10 to 100  in 0.22. | 100 |
|  | criterion criterion: {"squared\_error", "absolute\_error", "friedman\_mse", "poisson"}, default="squared\_error"  The function to measure the quality of a split. Supported criteria are "squared\_error" for the mean squared error, which is equal to variance reduction as feature selection criterion and minimizes the L2 loss using the mean of each terminal node, "friedman\_mse", which uses mean squared error with Friedman's improvement score for potential splits, "absolute\_error" for the mean absolute error, which minimizes the L1 loss using the median of each terminal node, and "poisson" which uses reduction in Poisson deviance to find splits. Training using "absolute\_error" is significantly slower than when using "squared\_error".  .. versionadded:: 0.18  Mean Absolute Error (MAE) criterion.  .. versionadded:: 1.0  Poisson criterion. | 'squared\_error' |
|  | max\_depth max\_depth: int, default=None  The maximum depth of the tree. If None, then nodes are expanded until all leaves are pure or until all leaves contain less than min\_samples\_split samples. | None |
|  | min\_samples\_split min\_samples\_split: int or float, default=2  The minimum number of samples required to split an internal node:  - If int, then consider `min\_samples\_split` as the minimum number. - If float, then `min\_samples\_split` is a fraction and  `ceil(min\_samples\_split \* n\_samples)` are the minimum  number of samples for each split.  .. versionchanged:: 0.18  Added float values for fractions. | 2 |
|  | min\_samples\_leaf min\_samples\_leaf: int or float, default=1  The minimum number of samples required to be at a leaf node. A split point at any depth will only be considered if it leaves at least ``min\_samples\_leaf`` training samples in each of the left and right branches. This may have the effect of smoothing the model, especially in regression.  - If int, then consider `min\_samples\_leaf` as the minimum number. - If float, then `min\_samples\_leaf` is a fraction and  `ceil(min\_samples\_leaf \* n\_samples)` are the minimum  number of samples for each node.  .. versionchanged:: 0.18  Added float values for fractions. | 1 |
|  | min\_weight\_fraction\_leaf min\_weight\_fraction\_leaf: float, default=0.0  The minimum weighted fraction of the sum total of weights (of all the input samples) required to be at a leaf node. Samples have equal weight when sample\_weight is not provided. | 0.0 |
|  | max\_features max\_features: {"sqrt", "log2", None}, int or float, default=1.0  The number of features to consider when looking for the best split:  - If int, then consider `max\_features` features at each split. - If float, then `max\_features` is a fraction and  `max(1, int(max\_features \* n\_features\_in\_))` features are considered at each  split. - If "sqrt", then `max\_features=sqrt(n\_features)`. - If "log2", then `max\_features=log2(n\_features)`. - If None or 1.0, then `max\_features=n\_features`.  .. note::  The default of 1.0 is equivalent to bagged trees and more  randomness can be achieved by setting smaller values, e.g. 0.3.  .. versionchanged:: 1.1  The default of `max\_features` changed from `"auto"` to 1.0.  Note: the search for a split does not stop until at least one valid partition of the node samples is found, even if it requires to effectively inspect more than ``max\_features`` features. | 1.0 |
|  | max\_leaf\_nodes max\_leaf\_nodes: int, default=None  Grow trees with ``max\_leaf\_nodes`` in best-first fashion. Best nodes are defined as relative reduction in impurity. If None then unlimited number of leaf nodes. | None |
|  | min\_impurity\_decrease min\_impurity\_decrease: float, default=0.0  A node will be split if this split induces a decrease of the impurity greater than or equal to this value.  The weighted impurity decrease equation is the following::   N\_t / N \* (impurity - N\_t\_R / N\_t \* right\_impurity  - N\_t\_L / N\_t \* left\_impurity)  where ``N`` is the total number of samples, ``N\_t`` is the number of samples at the current node, ``N\_t\_L`` is the number of samples in the left child, and ``N\_t\_R`` is the number of samples in the right child.  ``N``, ``N\_t``, ``N\_t\_R`` and ``N\_t\_L`` all refer to the weighted sum, if ``sample\_weight`` is passed.  .. versionadded:: 0.19 | 0.0 |
|  | bootstrap bootstrap: bool, default=True  Whether bootstrap samples are used when building trees. If False, the whole dataset is used to build each tree. | True |
|  | oob\_score oob\_score: bool or callable, default=False  Whether to use out-of-bag samples to estimate the generalization score. By default, :func:`~sklearn.metrics.r2\_score` is used. Provide a callable with signature `metric(y\_true, y\_pred)` to use a custom metric. Only available if `bootstrap=True`.  For an illustration of out-of-bag (OOB) error estimation, see the example :ref:`sphx\_glr\_auto\_examples\_ensemble\_plot\_ensemble\_oob.py`. | False |
|  | n\_jobs n\_jobs: int, default=None  The number of jobs to run in parallel. :meth:`fit`, :meth:`predict`, :meth:`decision\_path` and :meth:`apply` are all parallelized over the trees. ``None`` means 1 unless in a :obj:`joblib.parallel\_backend` context. ``-1`` means using all processors. See :term:`Glossary ` for more details. | -1 |
|  | random\_state random\_state: int, RandomState instance or None, default=None  Controls both the randomness of the bootstrapping of the samples used when building trees (if ``bootstrap=True``) and the sampling of the features to consider when looking for the best split at each node (if ``max\_features < n\_features``). See :term:`Glossary ` for details. | 42 |
|  | verbose verbose: int, default=0  Controls the verbosity when fitting and predicting. | 0 |
|  | warm\_start warm\_start: bool, default=False  When set to ``True``, reuse the solution of the previous call to fit and add more estimators to the ensemble, otherwise, just fit a whole new forest. See :term:`Glossary ` and :ref:`tree\_ensemble\_warm\_start` for details. | False |
|  | ccp\_alpha ccp\_alpha: non-negative float, default=0.0  Complexity parameter used for Minimal Cost-Complexity Pruning. The subtree with the largest cost complexity that is smaller than ``ccp\_alpha`` will be chosen. By default, no pruning is performed. See :ref:`minimal\_cost\_complexity\_pruning` for details. See :ref:`sphx\_glr\_auto\_examples\_tree\_plot\_cost\_complexity\_pruning.py` for an example of such pruning.  .. versionadded:: 0.22 | 0.0 |
|  | max\_samples max\_samples: int or float, default=None  If bootstrap is True, the number of samples to draw from X to train each base estimator.  - If None (default), then draw `X.shape[0]` samples. - If int, then draw `max\_samples` samples. - If float, then draw `max(round(n\_samples \* max\_samples), 1)` samples. Thus,  `max\_samples` should be in the interval `(0.0, 1.0]`.  .. versionadded:: 0.22 | None |
|  | monotonic\_cst monotonic\_cst: array-like of int of shape (n\_features), default=None  Indicates the monotonicity constraint to enforce on each feature.  - 1: monotonically increasing  - 0: no constraint  - -1: monotonically decreasing  If monotonic\_cst is None, no constraints are applied.  Monotonicity constraints are not supported for:  - multioutput regressions (i.e. when `n\_outputs\_ > 1`),  - regressions trained on data with missing values.  Read more in the :ref:`User Guide `.  .. versionadded:: 1.4 | None |

In [13]:

```
# Make predictions
y_pred_train_log = rf_model.predict(X_train)
y_pred_test_log = rf_model.predict(X_test)
```

### Evaluation¶

In [14]:

```
# Evaluate the model
print("\n=== Model Performance ===")
print("\nTraining Set:")
print(f"R² Score: {r2_score(y_train_log, y_pred_train_log):.4f}")
print(f"RMSE: {np.sqrt(mean_squared_error(y_train_log, y_pred_train_log)):.4f}")
print(f"MAE: {mean_absolute_error(y_train_log, y_pred_train_log):.4f}")

print("\nTest Set:")
print(f"R² Score: {r2_score(y_test_log, y_pred_test_log):.4f}")
print(f"RMSE: {np.sqrt(mean_squared_error(y_test_log, y_pred_test_log)):.4f}")
print(f"MAE: {mean_absolute_error(y_test_log, y_pred_test_log):.4f}")
```

```
=== Model Performance ===

Training Set:
R² Score: 0.8648
RMSE: 0.2025
MAE: 0.1386

Test Set:
R² Score: 0.2155
RMSE: 0.4847
MAE: 0.3273
```

In [15]:

```
# Plot predictions vs actual values
plt.figure(figsize=(10, 6))
plt.scatter(y_train_log, y_pred_train_log, alpha=0.3, color='green', label='Training set')
plt.scatter(y_test_log, y_pred_test_log, alpha=0.5, color='pink', label='Test set')

# Perfect prediction line (slope=1, intercept=0)
plt.plot([y_log.min(), y_log.max()], [y_log.min(), y_log.max()],
         'r--', lw=2, label='Perfect prediction')

# 10x error lines (slope=1, intercept=±1)
plt.plot([y_log.min(), y_log.max()], [y_log.min() + 1, y_log.max() + 1],
         'b--', lw=1.5, alpha=0.7, label='+10x error')
plt.plot([y_log.min(), y_log.max()], [y_log.min() - 1, y_log.max() - 1],
         'b--', lw=1.5, alpha=0.7, label='-10x error')

plt.xlabel('Actual log(fold_error)')
plt.ylabel('Predicted log(fold_error)')
plt.title('Actual vs Predicted log(fold_error)')
plt.legend()
plt.tight_layout()
plt.show()
```

The log(fold error) model show poor predictive power, therefore, we do not continue with feature importance analysis for this model.

## For log error predictions¶

#### Filter the data¶

In [16]:

```
df_filtered_log = df[df['sample'] == 'HPLC_high'].copy()

print("Filtered dataset shape:", df_filtered_log.shape)
```

```
Filtered dataset shape: (4449, 19)
```

In [17]:

```
# Select only the columns we need for features and target
feature_columns_log = ['compound', 'approach', 'analyzer', 'mobile_phase_add', 'org_phase', 
                   'inj_vol_ul', 'spray_volt_kv', 'grad_length_min']
target_column_log = 'log_error'
```

### Feature matrix¶

In [18]:

```
X = df_filtered_log[feature_columns_log].copy()
y = df_filtered_log[target_column_log].copy()
```

In [19]:

```
# Check for missing values
print("\nMissing values in features:")
print(X.isnull().sum())
print("\nMissing values in target:")
print(y.isnull().sum())
```

```
Missing values in features:
compound            0
approach            0
analyzer            0
mobile_phase_add    0
org_phase           0
inj_vol_ul          0
spray_volt_kv       0
grad_length_min     0
dtype: int64

Missing values in target:
0
```

In [20]:

```
# Display unique values for each categorical column
print("\n=== Unique values per feature ===")
for col in feature_columns_log:
    print(f"\n{col}: {X[col].nunique()} unique values")
    print(X[col].value_counts())
```

```
=== Unique values per feature ===

compound: 41 unique values
compound
2-methylbenzothiazole                       156
Atrazine-2-hydroxy                          156
Metolachlor-ESA                             153
Carbamazepine-10,11-epoxide                 151
Atrazine-desethyl                           150
Simazine-2-hydroxy                          149
5-methyl-1H-benzotriazole                   148
5-chlorobenzotriazole                       148
2-(methylthio)benzothiazole                 147
2-aminobenzothiazole                        146
Theophylline                                145
Atrazine-desisopropyl                       145
2-hydroxybenzothiazole                      144
Monuron                                     139
Metazachlor                                 136
Benzotriazole-5-carboxylic acid             134
Metolachlor-OA                              132
10,11-dihydro-10-hydroxycarbamazepine       126
Climbazole                                  124
Irgarol                                     121
Thiabendazole                               120
Omethoate                                   120
Phenazine                                   117
Methidathion                                116
Atrazine-desethyl-desisopropyl              112
Sudan I                                     109
Sebuthylazine                               109
Atrazine-desethyl-2-hydroxy                 101
Adenosine                                    96
Chlorpyrifos                                 89
Methomyl                                     77
Benzothiazole                                77
Acephate                                     72
Ketoconazole                                 68
Atrazine-desisopropyl-2-hydroxy              59
Naproxen                                     39
Melamine                                     35
Clotrimazole                                 32
Metformin                                    24
Atrazine-desethyl-desisopropyl-2-hydroxy     19
Reserpine                                     8
Name: count, dtype: int64

approach: 5 unique values
approach
close_elut    1011
struct_sim    1011
randfor_IE     964
MLR_IE         911
parent         552
Name: count, dtype: int64

analyzer: 2 unique values
analyzer
orb    2597
tof    1852
Name: count, dtype: int64

mobile_phase_add: 2 unique values
mobile_phase_add
acid      3317
buffer    1132
Name: count, dtype: int64

org_phase: 2 unique values
org_phase
meoh    2248
mecn    2201
Name: count, dtype: int64

inj_vol_ul: 3 unique values
inj_vol_ul
10_ul_below    2651
above_25_ul    1198
10_25_ul        600
Name: count, dtype: int64

spray_volt_kv: 3 unique values
spray_volt_kv
3.0_3.5_kv      2000
above_3.5_kv    1391
3.0_kv_below    1058
Name: count, dtype: int64

grad_length_min: 2 unique values
grad_length_min
above_25_min    2266
25_min_below    2183
Name: count, dtype: int64
```

### Encode categorical variables¶

- For 'compound', we'll use Label Encoding since it's the compound name
- For the other features, we can also use Label Encoding or One-Hot Encoding
- Let's use Label Encoding for all to keep it simple

In [21]:

```
label_encoders_log = {}
X_encoded_log = X.copy()

for col in feature_columns_log:
    le = LabelEncoder()
    X_encoded_log[col] = le.fit_transform(X[col].astype(str))
    label_encoders_log[col] = le
    print(f"\nEncoded {col}:")
    print(f"  Original values: {le.classes_}")
    print(f"  Encoded as: {list(range(len(le.classes_)))}")
```

```
Encoded compound:
  Original values: ['10,11-dihydro-10-hydroxycarbamazepine' '2-(methylthio)benzothiazole'
 '2-aminobenzothiazole' '2-hydroxybenzothiazole' '2-methylbenzothiazole'
 '5-chlorobenzotriazole' '5-methyl-1H-benzotriazole' 'Acephate'
 'Adenosine' 'Atrazine-2-hydroxy' 'Atrazine-desethyl'
 'Atrazine-desethyl-2-hydroxy' 'Atrazine-desethyl-desisopropyl'
 'Atrazine-desethyl-desisopropyl-2-hydroxy' 'Atrazine-desisopropyl'
 'Atrazine-desisopropyl-2-hydroxy' 'Benzothiazole'
 'Benzotriazole-5-carboxylic acid' 'Carbamazepine-10,11-epoxide'
 'Chlorpyrifos' 'Climbazole' 'Clotrimazole' 'Irgarol' 'Ketoconazole'
 'Melamine' 'Metazachlor' 'Metformin' 'Methidathion' 'Methomyl'
 'Metolachlor-ESA' 'Metolachlor-OA' 'Monuron' 'Naproxen' 'Omethoate'
 'Phenazine' 'Reserpine' 'Sebuthylazine' 'Simazine-2-hydroxy' 'Sudan I'
 'Theophylline' 'Thiabendazole']
  Encoded as: [0, 1, 2, 3, 4, 5, 6, 7, 8, 9, 10, 11, 12, 13, 14, 15, 16, 17, 18, 19, 20, 21, 22, 23, 24, 25, 26, 27, 28, 29, 30, 31, 32, 33, 34, 35, 36, 37, 38, 39, 40]

Encoded approach:
  Original values: ['MLR_IE' 'close_elut' 'parent' 'randfor_IE' 'struct_sim']
  Encoded as: [0, 1, 2, 3, 4]

Encoded analyzer:
  Original values: ['orb' 'tof']
  Encoded as: [0, 1]

Encoded mobile_phase_add:
  Original values: ['acid' 'buffer']
  Encoded as: [0, 1]

Encoded org_phase:
  Original values: ['mecn' 'meoh']
  Encoded as: [0, 1]

Encoded inj_vol_ul:
  Original values: ['10_25_ul' '10_ul_below' 'above_25_ul']
  Encoded as: [0, 1, 2]

Encoded spray_volt_kv:
  Original values: ['3.0_3.5_kv' '3.0_kv_below' 'above_3.5_kv']
  Encoded as: [0, 1, 2]

Encoded grad_length_min:
  Original values: ['25_min_below' 'above_25_min']
  Encoded as: [0, 1]
```

### Random forest¶

In [22]:

```
# Use 'dataset' as the grouping variable for splitting
groups = df_filtered_log['dataset']

# GroupShuffleSplit ensures all samples from a dataset go to either train or test
gss = GroupShuffleSplit(n_splits=1, test_size=0.2, random_state=42)

# Get the train/test indices
train_idx, test_idx = next(gss.split(X_encoded_log, y, groups=groups))

# Split the data
X_train_log = X_encoded_log.iloc[train_idx]
X_test_log = X_encoded_log.iloc[test_idx]
y_train_log = y.iloc[train_idx]
y_test_log = y.iloc[test_idx]

print(f"\nTraining set size: {X_train_log.shape[0]}")
print(f"Test set size: {X_test_log.shape[0]}")
```

```
Training set size: 3407
Test set size: 1042
```

In [23]:

```
# Show which datasets ended up in train vs test
print(f"\nDatasets in training set: {df_filtered_log['dataset'].iloc[train_idx].unique()}")
print(f"Datasets in test set: {df_filtered_log['dataset'].iloc[test_idx].unique()}")
```

```
Datasets in training set: ['L1' 'L11' 'L12' 'L15' 'L17' 'L19' 'L21' 'L22' 'L25' 'L29' 'L3' 'L32'
 'L33' 'L34' 'L37' 'L38' 'L40' 'L41' 'L6' 'L7' 'L10' 'L18' 'L24' 'L26'
 'L5' 'L9' 'L28' 'L20' 'L31']
Datasets in test set: ['L16' 'L23' 'L27' 'L35' 'L36' 'L8' 'L14' 'L39']
```

In [24]:

```
# Initialize and train the Random Forest model
rf_model_log = RandomForestRegressor(
    n_estimators=100,
    max_depth=None,
    min_samples_split=2,
    min_samples_leaf=1,
    random_state=42,
    n_jobs=-1
)
```

In [25]:

```
# Train the model
print("\nTraining the model...")
rf_model_log.fit(X_train_log, y_train_log)
```

```
Training the model...
```

Out[25]:

```
RandomForestRegressor(n_jobs=-1, random_state=42)
```

**In a Jupyter environment, please rerun this cell to show the HTML representation or trust the notebook.   
On GitHub, the HTML representation is unable to render, please try loading this page with nbviewer.org.**

RandomForestRegressor

?Documentation for RandomForestRegressoriFitted

Parameters

|  |  |  |
| --- | --- | --- |
|  | n\_estimators n\_estimators: int, default=100  The number of trees in the forest.  .. versionchanged:: 0.22  The default value of ``n\_estimators`` changed from 10 to 100  in 0.22. | 100 |
|  | criterion criterion: {"squared\_error", "absolute\_error", "friedman\_mse", "poisson"}, default="squared\_error"  The function to measure the quality of a split. Supported criteria are "squared\_error" for the mean squared error, which is equal to variance reduction as feature selection criterion and minimizes the L2 loss using the mean of each terminal node, "friedman\_mse", which uses mean squared error with Friedman's improvement score for potential splits, "absolute\_error" for the mean absolute error, which minimizes the L1 loss using the median of each terminal node, and "poisson" which uses reduction in Poisson deviance to find splits. Training using "absolute\_error" is significantly slower than when using "squared\_error".  .. versionadded:: 0.18  Mean Absolute Error (MAE) criterion.  .. versionadded:: 1.0  Poisson criterion. | 'squared\_error' |
|  | max\_depth max\_depth: int, default=None  The maximum depth of the tree. If None, then nodes are expanded until all leaves are pure or until all leaves contain less than min\_samples\_split samples. | None |
|  | min\_samples\_split min\_samples\_split: int or float, default=2  The minimum number of samples required to split an internal node:  - If int, then consider `min\_samples\_split` as the minimum number. - If float, then `min\_samples\_split` is a fraction and  `ceil(min\_samples\_split \* n\_samples)` are the minimum  number of samples for each split.  .. versionchanged:: 0.18  Added float values for fractions. | 2 |
|  | min\_samples\_leaf min\_samples\_leaf: int or float, default=1  The minimum number of samples required to be at a leaf node. A split point at any depth will only be considered if it leaves at least ``min\_samples\_leaf`` training samples in each of the left and right branches. This may have the effect of smoothing the model, especially in regression.  - If int, then consider `min\_samples\_leaf` as the minimum number. - If float, then `min\_samples\_leaf` is a fraction and  `ceil(min\_samples\_leaf \* n\_samples)` are the minimum  number of samples for each node.  .. versionchanged:: 0.18  Added float values for fractions. | 1 |
|  | min\_weight\_fraction\_leaf min\_weight\_fraction\_leaf: float, default=0.0  The minimum weighted fraction of the sum total of weights (of all the input samples) required to be at a leaf node. Samples have equal weight when sample\_weight is not provided. | 0.0 |
|  | max\_features max\_features: {"sqrt", "log2", None}, int or float, default=1.0  The number of features to consider when looking for the best split:  - If int, then consider `max\_features` features at each split. - If float, then `max\_features` is a fraction and  `max(1, int(max\_features \* n\_features\_in\_))` features are considered at each  split. - If "sqrt", then `max\_features=sqrt(n\_features)`. - If "log2", then `max\_features=log2(n\_features)`. - If None or 1.0, then `max\_features=n\_features`.  .. note::  The default of 1.0 is equivalent to bagged trees and more  randomness can be achieved by setting smaller values, e.g. 0.3.  .. versionchanged:: 1.1  The default of `max\_features` changed from `"auto"` to 1.0.  Note: the search for a split does not stop until at least one valid partition of the node samples is found, even if it requires to effectively inspect more than ``max\_features`` features. | 1.0 |
|  | max\_leaf\_nodes max\_leaf\_nodes: int, default=None  Grow trees with ``max\_leaf\_nodes`` in best-first fashion. Best nodes are defined as relative reduction in impurity. If None then unlimited number of leaf nodes. | None |
|  | min\_impurity\_decrease min\_impurity\_decrease: float, default=0.0  A node will be split if this split induces a decrease of the impurity greater than or equal to this value.  The weighted impurity decrease equation is the following::   N\_t / N \* (impurity - N\_t\_R / N\_t \* right\_impurity  - N\_t\_L / N\_t \* left\_impurity)  where ``N`` is the total number of samples, ``N\_t`` is the number of samples at the current node, ``N\_t\_L`` is the number of samples in the left child, and ``N\_t\_R`` is the number of samples in the right child.  ``N``, ``N\_t``, ``N\_t\_R`` and ``N\_t\_L`` all refer to the weighted sum, if ``sample\_weight`` is passed.  .. versionadded:: 0.19 | 0.0 |
|  | bootstrap bootstrap: bool, default=True  Whether bootstrap samples are used when building trees. If False, the whole dataset is used to build each tree. | True |
|  | oob\_score oob\_score: bool or callable, default=False  Whether to use out-of-bag samples to estimate the generalization score. By default, :func:`~sklearn.metrics.r2\_score` is used. Provide a callable with signature `metric(y\_true, y\_pred)` to use a custom metric. Only available if `bootstrap=True`.  For an illustration of out-of-bag (OOB) error estimation, see the example :ref:`sphx\_glr\_auto\_examples\_ensemble\_plot\_ensemble\_oob.py`. | False |
|  | n\_jobs n\_jobs: int, default=None  The number of jobs to run in parallel. :meth:`fit`, :meth:`predict`, :meth:`decision\_path` and :meth:`apply` are all parallelized over the trees. ``None`` means 1 unless in a :obj:`joblib.parallel\_backend` context. ``-1`` means using all processors. See :term:`Glossary ` for more details. | -1 |
|  | random\_state random\_state: int, RandomState instance or None, default=None  Controls both the randomness of the bootstrapping of the samples used when building trees (if ``bootstrap=True``) and the sampling of the features to consider when looking for the best split at each node (if ``max\_features < n\_features``). See :term:`Glossary ` for details. | 42 |
|  | verbose verbose: int, default=0  Controls the verbosity when fitting and predicting. | 0 |
|  | warm\_start warm\_start: bool, default=False  When set to ``True``, reuse the solution of the previous call to fit and add more estimators to the ensemble, otherwise, just fit a whole new forest. See :term:`Glossary ` and :ref:`tree\_ensemble\_warm\_start` for details. | False |
|  | ccp\_alpha ccp\_alpha: non-negative float, default=0.0  Complexity parameter used for Minimal Cost-Complexity Pruning. The subtree with the largest cost complexity that is smaller than ``ccp\_alpha`` will be chosen. By default, no pruning is performed. See :ref:`minimal\_cost\_complexity\_pruning` for details. See :ref:`sphx\_glr\_auto\_examples\_tree\_plot\_cost\_complexity\_pruning.py` for an example of such pruning.  .. versionadded:: 0.22 | 0.0 |
|  | max\_samples max\_samples: int or float, default=None  If bootstrap is True, the number of samples to draw from X to train each base estimator.  - If None (default), then draw `X.shape[0]` samples. - If int, then draw `max\_samples` samples. - If float, then draw `max(round(n\_samples \* max\_samples), 1)` samples. Thus,  `max\_samples` should be in the interval `(0.0, 1.0]`.  .. versionadded:: 0.22 | None |
|  | monotonic\_cst monotonic\_cst: array-like of int of shape (n\_features), default=None  Indicates the monotonicity constraint to enforce on each feature.  - 1: monotonically increasing  - 0: no constraint  - -1: monotonically decreasing  If monotonic\_cst is None, no constraints are applied.  Monotonicity constraints are not supported for:  - multioutput regressions (i.e. when `n\_outputs\_ > 1`),  - regressions trained on data with missing values.  Read more in the :ref:`User Guide `.  .. versionadded:: 1.4 | None |

In [26]:

```
# Make predictions
y_pred_train_log = rf_model_log.predict(X_train_log)
y_pred_test_log = rf_model_log.predict(X_test_log)
```

### Evaluation¶

In [27]:

```
# Evaluate the model
print("\n=== Model Performance ===")
print("\nTraining Set:")
print(f"R² Score: {r2_score(y_train_log, y_pred_train_log):.4f}")
print(f"RMSE: {np.sqrt(mean_squared_error(y_train_log, y_pred_train_log)):.4f}")
print(f"MAE: {mean_absolute_error(y_train_log, y_pred_train_log):.4f}")

print("\nTest Set:")
print(f"R² Score: {r2_score(y_test_log, y_pred_test_log):.4f}")
print(f"RMSE: {np.sqrt(mean_squared_error(y_test_log, y_pred_test_log)):.4f}")
print(f"MAE: {mean_absolute_error(y_test_log, y_pred_test_log):.4f}")
```

```
=== Model Performance ===

Training Set:
R² Score: 0.9078
RMSE: 0.2496
MAE: 0.1633

Test Set:
R² Score: 0.3305
RMSE: 0.6564
MAE: 0.4216
```

In [28]:

```
# Plot predictions vs actual values
plt.figure(figsize=(10, 6))
plt.scatter(y_train_log, y_pred_train_log, alpha=0.3, color='green', label='Training set')
plt.scatter(y_test_log, y_pred_test_log, alpha=0.5, color='pink', label='Test set')

# Perfect prediction line (slope=1, intercept=0)
plt.plot([y.min(), y.max()], [y.min(), y.max()], 
         'r--', lw=2, label='Perfect prediction')

# 10x error lines (slope=1, intercept=±1)
plt.plot([y.min(), y.max()], [y.min() + 1, y.max() + 1], 
         'b--', lw=1.5, alpha=0.7, label='+10x error')
plt.plot([y.min(), y.max()], [y.min() - 1, y.max() - 1], 
         'b--', lw=1.5, alpha=0.7, label='-10x error')


plt.xlabel('Actual log(error)')
plt.ylabel('Predicted log(error)')
plt.title('Actual vs Predicted log(error)')
plt.legend()
plt.tight_layout()
plt.show()
```

### Evaluate the feature importance with SHAP¶

In [29]:

```
# Create a SHAP explainer for the Random Forest model
explainer_log = shap.TreeExplainer(rf_model_log)

# Calculate SHAP values for the test set
shap_values_log = explainer_log.shap_values(X_test_log)
```

### Plots¶

In [30]:

```
# Summary plot (beeswarm) - shows feature importance and direction of effect
shap.summary_plot(shap_values_log, X_test_log, feature_names=feature_columns_log)
```

#### Beeswarm plot without the 'compound' feature¶

In [31]:

```
# Get the index of 'compound' in the feature list
compound_idx = feature_columns_log.index('compound')

# Remove 'compound' from the feature names
feature_columns_no_compound = [f for f in feature_columns_log if f != 'compound']

# Remove the corresponding column from SHAP values and X_test
shap_values_no_compound = np.delete(shap_values_log, compound_idx, axis=1)
X_test_no_compound = X_test_log.drop(columns='compound')

# Beeswarm plot without 'compound'
shap.summary_plot(shap_values_no_compound, X_test_no_compound, 
                  feature_names=feature_columns_no_compound)
```

#### Beeswarm plot without the 'compound' and 'approach' features¶

In [32]:

```
# Get the indices of 'compound' and 'approach' in the feature list
compound_idx = feature_columns_log.index('compound')
approach_idx = feature_columns_log.index('approach')

# Remove 'compound' and 'approach' from the feature names
feature_columns_filtered = [f for f in feature_columns_log if f not in ['compound', 'approach']]

# Remove the corresponding columns from SHAP values and X_test
# Note: Remove indices in descending order to avoid index shifting issues
indices_to_remove = sorted([compound_idx, approach_idx], reverse=True)
shap_values_filtered = shap_values_log.copy()
for idx in indices_to_remove:
    shap_values_filtered = np.delete(shap_values_filtered, idx, axis=1)

X_test_filtered = X_test_log.drop(columns=['compound', 'approach'])

# Beeswarm plot without 'compound' and 'approach'
shap.summary_plot(shap_values_filtered, X_test_filtered, 
                  feature_names=feature_columns_filtered)
```

#### Bar plot¶

In [33]:

```
# Bar plot - shows mean absolute SHAP values (overall importance ranking)
shap.summary_plot(shap_values_log, X_test_log, feature_names=feature_columns_log, plot_type='bar')
```
