# Supplementary material for: In-Depth Analysis of the Data from an Interlaboratory Study of Quantitative Non-Target Screening—How Do the Instrumental Methods Compare?
Source: Molecules. 2026 Mar 6;31(5):875. doi: 10.3390/molecules31050875 (PMC12986245; doi:10.3390/molecules31050875)
Supplement: Supplementary file 1 [file molecules-31-00875-s001.zip › Code S3_logRF_projection_analysis.html]

RF projection between labs with GAM


# RF projection between labs with GAM

#### Anneli Kruve & Louise Malm

## Read in the libraries

```
library(caret)
library(Metrics)
library(mgcv)
library(rstatix)
library(tidyverse)
library(plotly)
source("theme.R")
```

## Read in the data

Experimental RF data from the interlaboratory comparison. (L2 was
removed since it only had values in one of the water matrices, and only
in the low concentrated spiked sample)

```
data = read_delim("RF_data.csv",
                  delim = ",",
                  col_names = T) %>% 
  filter(lab != "L2")
```

## Anchoring logRF values

Data preprocessing for creating all-to-L38 combinations.

```
data_all = data %>% 
  select(compound, SMILES, type, lab, RF, logRF, RT)

data_SU = data %>%
  filter(lab == "L38") %>%
  select(compound, SMILES, type, lab_SU = lab, RF_SU = RF, logRF_SU = logRF, RT_SU = RT)

data_all_SU = data_all %>% 
  left_join(data_SU)
```

Performing GAM

```
data_all_SU_pred = tibble()

for(lab_this in levels(factor(data_all_SU$lab))) {
  print(lab_this)
  data_xy_this_that = data_all_SU %>%
    filter(lab == lab_this) %>%
    select(lab, compound, logRF, logRF_SU, type, RT) %>%
    unique()

    model_this = gam(logRF_SU ~ s(logRF, bs = "cr", k = 6),
                     data = data_xy_this_that %>%
                       filter(type == "cals"))

    data_xy_this_that = data_xy_this_that %>%
      mutate(logRF_pred = predict.gam(model_this,
                                        newdata = data_xy_this_that))

    data_all_SU_pred = data_all_SU_pred %>%
      bind_rows(data_xy_this_that)
    
    min_val <- min(data_xy_this_that$logRF, data_xy_this_that$logRF_SU, data_xy_this_that$logRF_pred, na.rm = T)
    max_val <- max(data_xy_this_that$logRF, data_xy_this_that$logRF_SU, data_xy_this_that$logRF_pred, na.rm = T)

  print(
    ggplot(data = data_all_SU_pred %>%
             filter(lab == lab_this & type == "sus")) +
      geom_point(mapping = aes(x = logRF,
                               y = logRF_SU),
                 color = "#659D8B",
                 size = 3,
                 alpha = 0.8) +
      geom_point(mapping = aes(x = logRF_pred,
                               y = logRF_SU),
                 color = "#8C2155",
                 size = 3,
                 alpha = 0.8) +
      geom_abline(intercept = 0, slope = 1) +
      scale_x_continuous(limits = c(min_val, max_val)) +
      scale_y_continuous(limits = c(min_val, max_val)) +
      labs(x = "projection", y = "original", title = lab_this) +
      my_theme +
      theme(axis.text.x = element_text(angle = 90))
  )
}
```

```
## [1] "L1"
```

```
## Warning: Removed 1 row containing missing values or values outside the scale range
## (`geom_point()`).
## Removed 1 row containing missing values or values outside the scale range
## (`geom_point()`).
```

```
## [1] "L10"
```

```
## Warning: Removed 7 rows containing missing values or values outside the scale range
## (`geom_point()`).
```

```
## Warning: Removed 7 rows containing missing values or values outside the scale range
## (`geom_point()`).
```

```
## [1] "L11"
```

```
## Warning: Removed 2 rows containing missing values or values outside the scale range
## (`geom_point()`).
```

```
## Warning: Removed 2 rows containing missing values or values outside the scale range
## (`geom_point()`).
```

```
## [1] "L12"
```

```
## Warning: Removed 1 row containing missing values or values outside the scale range
## (`geom_point()`).
```

```
## Warning: Removed 1 row containing missing values or values outside the scale range
## (`geom_point()`).
```

```
## [1] "L14"
```

```
## Warning: Removed 4 rows containing missing values or values outside the scale range
## (`geom_point()`).
```

```
## Warning: Removed 4 rows containing missing values or values outside the scale range
## (`geom_point()`).
```

```
## [1] "L15"
```

```
## Warning: Removed 3 rows containing missing values or values outside the scale range
## (`geom_point()`).
```

```
## Warning: Removed 3 rows containing missing values or values outside the scale range
## (`geom_point()`).
```

```
## [1] "L16"
```

```
## Warning: Removed 2 rows containing missing values or values outside the scale range
## (`geom_point()`).
```

```
## Warning: Removed 2 rows containing missing values or values outside the scale range
## (`geom_point()`).
```

```
## [1] "L17"
```

```
## Warning: Removed 4 rows containing missing values or values outside the scale range
## (`geom_point()`).
```

```
## Warning: Removed 4 rows containing missing values or values outside the scale range
## (`geom_point()`).
```

```
## [1] "L18"
```

```
## Warning: Removed 2 rows containing missing values or values outside the scale range
## (`geom_point()`).
```

```
## Warning: Removed 2 rows containing missing values or values outside the scale range
## (`geom_point()`).
```

```
## [1] "L19"
```

```
## Warning: Removed 4 rows containing missing values or values outside the scale range
## (`geom_point()`).
```

```
## Warning: Removed 4 rows containing missing values or values outside the scale range
## (`geom_point()`).
```

```
## [1] "L20"
```

```
## Warning: Removed 1 row containing missing values or values outside the scale range
## (`geom_point()`).
```

```
## Warning: Removed 1 row containing missing values or values outside the scale range
## (`geom_point()`).
```

```
## [1] "L21"
```

```
## Warning: Removed 2 rows containing missing values or values outside the scale range
## (`geom_point()`).
```

```
## Warning: Removed 2 rows containing missing values or values outside the scale range
## (`geom_point()`).
```

```
## [1] "L22"
```

```
## Warning: Removed 2 rows containing missing values or values outside the scale range
## (`geom_point()`).
## Removed 2 rows containing missing values or values outside the scale range
## (`geom_point()`).
```

```
## [1] "L23"
```

```
## Warning: Removed 6 rows containing missing values or values outside the scale range
## (`geom_point()`).
```

```
## Warning: Removed 6 rows containing missing values or values outside the scale range
## (`geom_point()`).
```

```
## [1] "L24"
```

```
## Warning: Removed 2 rows containing missing values or values outside the scale range
## (`geom_point()`).
```

```
## Warning: Removed 2 rows containing missing values or values outside the scale range
## (`geom_point()`).
```

```
## [1] "L25"
```

```
## Warning: Removed 3 rows containing missing values or values outside the scale range
## (`geom_point()`).
```

```
## Warning: Removed 3 rows containing missing values or values outside the scale range
## (`geom_point()`).
```

```
## [1] "L26"
```

```
## Warning: Removed 2 rows containing missing values or values outside the scale range
## (`geom_point()`).
```

```
## Warning: Removed 2 rows containing missing values or values outside the scale range
## (`geom_point()`).
```

```
## [1] "L27"
```

```
## Warning: Removed 3 rows containing missing values or values outside the scale range
## (`geom_point()`).
```

```
## Warning: Removed 3 rows containing missing values or values outside the scale range
## (`geom_point()`).
```

```
## [1] "L28"
```

```
## Warning: Removed 1 row containing missing values or values outside the scale range
## (`geom_point()`).
```

```
## Warning: Removed 1 row containing missing values or values outside the scale range
## (`geom_point()`).
```

```
## [1] "L29"
```

```
## Warning: Removed 2 rows containing missing values or values outside the scale range
## (`geom_point()`).
```

```
## Warning: Removed 2 rows containing missing values or values outside the scale range
## (`geom_point()`).
```

```
## [1] "L3"
```

```
## Warning: Removed 3 rows containing missing values or values outside the scale range
## (`geom_point()`).
```

```
## Warning: Removed 3 rows containing missing values or values outside the scale range
## (`geom_point()`).
```

```
## [1] "L31"
```

```
## Warning: Removed 2 rows containing missing values or values outside the scale range
## (`geom_point()`).
```

```
## Warning: Removed 2 rows containing missing values or values outside the scale range
## (`geom_point()`).
```

```
## [1] "L32"
```

```
## Warning: Removed 3 rows containing missing values or values outside the scale range
## (`geom_point()`).
```

```
## Warning: Removed 3 rows containing missing values or values outside the scale range
## (`geom_point()`).
```

```
## [1] "L33"
```

```
## [1] "L34"
```

```
## Warning: Removed 5 rows containing missing values or values outside the scale range
## (`geom_point()`).
```

```
## Warning: Removed 5 rows containing missing values or values outside the scale range
## (`geom_point()`).
```

```
## [1] "L35"
```

```
## Warning: Removed 3 rows containing missing values or values outside the scale range
## (`geom_point()`).
```

```
## Warning: Removed 3 rows containing missing values or values outside the scale range
## (`geom_point()`).
```

```
## [1] "L36"
```

```
## Warning: Removed 3 rows containing missing values or values outside the scale range
## (`geom_point()`).
## Removed 3 rows containing missing values or values outside the scale range
## (`geom_point()`).
```

```
## [1] "L37"
```

```
## Warning: Removed 3 rows containing missing values or values outside the scale range
## (`geom_point()`).
## Removed 3 rows containing missing values or values outside the scale range
## (`geom_point()`).
```

```
## [1] "L38"
```

```
## [1] "L39"
```

```
## Warning: Removed 6 rows containing missing values or values outside the scale range
## (`geom_point()`).
```

```
## Warning: Removed 6 rows containing missing values or values outside the scale range
## (`geom_point()`).
```

```
## [1] "L40"
```

```
## Warning: Removed 3 rows containing missing values or values outside the scale range
## (`geom_point()`).
```

```
## Warning: Removed 3 rows containing missing values or values outside the scale range
## (`geom_point()`).
```

```
## [1] "L41"
```

```
## Warning: Removed 6 rows containing missing values or values outside the scale range
## (`geom_point()`).
```

```
## Warning: Removed 6 rows containing missing values or values outside the scale range
## (`geom_point()`).
```

```
## [1] "L5"
```

```
## Warning: Removed 3 rows containing missing values or values outside the scale range
## (`geom_point()`).
```

```
## Warning: Removed 3 rows containing missing values or values outside the scale range
## (`geom_point()`).
```

```
## [1] "L6"
```

```
## Warning: Removed 7 rows containing missing values or values outside the scale range
## (`geom_point()`).
```

```
## Warning: Removed 7 rows containing missing values or values outside the scale range
## (`geom_point()`).
```

```
## [1] "L7"
```

```
## Warning: Removed 2 rows containing missing values or values outside the scale range
## (`geom_point()`).
```

```
## Warning: Removed 2 rows containing missing values or values outside the scale range
## (`geom_point()`).
```

```
## [1] "L8"
```

```
## Warning: Removed 2 rows containing missing values or values outside the scale range
## (`geom_point()`).
## Removed 2 rows containing missing values or values outside the scale range
## (`geom_point()`).
```

```
## [1] "L9"
```

```
## Warning: Removed 2 rows containing missing values or values outside the scale range
## (`geom_point()`).
## Removed 2 rows containing missing values or values outside the scale range
## (`geom_point()`).
```

GAM but plotting both suspects and cals

```
for(lab_this in levels(factor(data_all_SU$lab))) {
  print(lab_this)

  print(
    ggplot(data = data_all_SU_pred %>%
             filter(lab == lab_this)) +
      geom_point(mapping = aes(x = logRF_pred,
                               y = logRF_SU,
                               color = type),
                 size = 3,
                 alpha = 0.8) +
      geom_abline(intercept = 0, slope = 1) +
      scale_x_continuous(limits = c(min_val, max_val)) +
      scale_y_continuous(limits = c(min_val, max_val)) +
      scale_color_manual(values = c("#659D8B", "#8C2155")) +
      labs(x = "projection", y = "orbitrap SU", title = lab_this) +
      my_theme +
      theme(axis.text.x = element_text(angle = 90))
  )
}
```

```
## [1] "L1"
```

```
## Warning: Removed 2 rows containing missing values or values outside the scale range
## (`geom_point()`).
```

```
## [1] "L10"
```

```
## Warning: Removed 12 rows containing missing values or values outside the scale range
## (`geom_point()`).
```

```
## [1] "L11"
```

```
## Warning: Removed 4 rows containing missing values or values outside the scale range
## (`geom_point()`).
```

```
## [1] "L12"
```

```
## Warning: Removed 1 row containing missing values or values outside the scale range
## (`geom_point()`).
```

```
## [1] "L14"
```

```
## Warning: Removed 8 rows containing missing values or values outside the scale range
## (`geom_point()`).
```

```
## [1] "L15"
```

```
## Warning: Removed 5 rows containing missing values or values outside the scale range
## (`geom_point()`).
```

```
## [1] "L16"
```

```
## Warning: Removed 5 rows containing missing values or values outside the scale range
## (`geom_point()`).
```

```
## [1] "L17"
```

```
## Warning: Removed 5 rows containing missing values or values outside the scale range
## (`geom_point()`).
```

```
## [1] "L18"
```

```
## Warning: Removed 4 rows containing missing values or values outside the scale range
## (`geom_point()`).
```

```
## [1] "L19"
```

```
## Warning: Removed 10 rows containing missing values or values outside the scale range
## (`geom_point()`).
```

```
## [1] "L20"
```

```
## Warning: Removed 3 rows containing missing values or values outside the scale range
## (`geom_point()`).
```

```
## [1] "L21"
```

```
## Warning: Removed 3 rows containing missing values or values outside the scale range
## (`geom_point()`).
```

```
## [1] "L22"
```

```
## Warning: Removed 3 rows containing missing values or values outside the scale range
## (`geom_point()`).
```

```
## [1] "L23"
```

```
## Warning: Removed 12 rows containing missing values or values outside the scale range
## (`geom_point()`).
```

```
## [1] "L24"
```

```
## Warning: Removed 6 rows containing missing values or values outside the scale range
## (`geom_point()`).
```

```
## [1] "L25"
```

```
## Warning: Removed 11 rows containing missing values or values outside the scale range
## (`geom_point()`).
```

```
## [1] "L26"
```

```
## Warning: Removed 4 rows containing missing values or values outside the scale range
## (`geom_point()`).
```

```
## [1] "L27"
```

```
## Warning: Removed 5 rows containing missing values or values outside the scale range
## (`geom_point()`).
```

```
## [1] "L28"
```

```
## Warning: Removed 2 rows containing missing values or values outside the scale range
## (`geom_point()`).
```

```
## [1] "L29"
```

```
## Warning: Removed 7 rows containing missing values or values outside the scale range
## (`geom_point()`).
```

```
## [1] "L3"
```

```
## Warning: Removed 4 rows containing missing values or values outside the scale range
## (`geom_point()`).
```

```
## [1] "L31"
```

```
## Warning: Removed 4 rows containing missing values or values outside the scale range
## (`geom_point()`).
```

```
## [1] "L32"
```

```
## Warning: Removed 8 rows containing missing values or values outside the scale range
## (`geom_point()`).
```

```
## [1] "L33"
```

```
## Warning: Removed 1 row containing missing values or values outside the scale range
## (`geom_point()`).
```

```
## [1] "L34"
```

```
## Warning: Removed 10 rows containing missing values or values outside the scale range
## (`geom_point()`).
```

```
## [1] "L35"
```

```
## Warning: Removed 5 rows containing missing values or values outside the scale range
## (`geom_point()`).
```

```
## [1] "L36"
```

```
## Warning: Removed 9 rows containing missing values or values outside the scale range
## (`geom_point()`).
```

```
## [1] "L37"
```

```
## Warning: Removed 5 rows containing missing values or values outside the scale range
## (`geom_point()`).
```

```
## [1] "L38"
```

```
## Warning: Removed 1 row containing missing values or values outside the scale range
## (`geom_point()`).
```

```
## [1] "L39"
```

```
## Warning: Removed 11 rows containing missing values or values outside the scale range
## (`geom_point()`).
```

```
## [1] "L40"
```

```
## Warning: Removed 6 rows containing missing values or values outside the scale range
## (`geom_point()`).
```

```
## [1] "L41"
```

```
## Warning: Removed 10 rows containing missing values or values outside the scale range
## (`geom_point()`).
```

```
## [1] "L5"
```

```
## Warning: Removed 5 rows containing missing values or values outside the scale range
## (`geom_point()`).
```

```
## [1] "L6"
```

```
## Warning: Removed 14 rows containing missing values or values outside the scale range
## (`geom_point()`).
```

```
## [1] "L7"
```

```
## Warning: Removed 3 rows containing missing values or values outside the scale range
## (`geom_point()`).
```

```
## [1] "L8"
```

```
## Warning: Removed 6 rows containing missing values or values outside the scale range
## (`geom_point()`).
```

```
## [1] "L9"
```

```
## Warning: Removed 3 rows containing missing values or values outside the scale range
## (`geom_point()`).
```

Linear model

```
data_all_SU_pred_lm = tibble()

for(lab_this in levels(factor(data_all_SU$lab))) {
  print(lab_this)
  data_xy_this_that = data_all_SU %>%
    filter(lab == lab_this) %>%
    select(lab, compound, logRF, logRF_SU, type, RT) %>%
    unique()

    model_this_lm = lm(logRF_SU ~ logRF,
                       data = data_xy_this_that %>%
                         filter(type == "cals"))

    data_xy_this_that = data_xy_this_that %>%
      mutate(logRF_pred = predict(model_this_lm,
                                  newdata = data_xy_this_that))

    data_all_SU_pred_lm = data_all_SU_pred_lm %>%
      bind_rows(data_xy_this_that)
    
    min_val <- min(data_xy_this_that$logRF, data_xy_this_that$logRF_SU, data_xy_this_that$logRF_pred, na.rm = T)
    max_val <- max(data_xy_this_that$logRF, data_xy_this_that$logRF_SU, data_xy_this_that$logRF_pred, na.rm = T)

  print(
    ggplot(data = data_all_SU_pred_lm %>%
             filter(lab == lab_this & type == "sus")) +
      geom_point(mapping = aes(x = logRF,
                               y = logRF_SU),
                 color = "#659D8B",
                 size = 3,
                 alpha = 0.8) +
      geom_point(mapping = aes(x = logRF_pred,
                               y = logRF_SU),
                 color = "#8C2155",
                 size = 3,
                 alpha = 0.8) +
      geom_abline(intercept = 0, slope = 1) +
      scale_x_continuous(limits = c(min_val, max_val)) +
      scale_y_continuous(limits = c(min_val, max_val)) +
      labs(x = "linear regression fit", y = "original", title = lab_this) +
      my_theme +
      theme(axis.text.x = element_text(angle = 90))
  )
}
```

```
## [1] "L1"
```

```
## Warning: Removed 1 row containing missing values or values outside the scale range
## (`geom_point()`).
## Removed 1 row containing missing values or values outside the scale range
## (`geom_point()`).
```

```
## [1] "L10"
```

```
## Warning: Removed 7 rows containing missing values or values outside the scale range
## (`geom_point()`).
```

```
## Warning: Removed 7 rows containing missing values or values outside the scale range
## (`geom_point()`).
```

```
## [1] "L11"
```

```
## Warning: Removed 2 rows containing missing values or values outside the scale range
## (`geom_point()`).
```

```
## Warning: Removed 2 rows containing missing values or values outside the scale range
## (`geom_point()`).
```

```
## [1] "L12"
```

```
## Warning: Removed 1 row containing missing values or values outside the scale range
## (`geom_point()`).
```

```
## Warning: Removed 1 row containing missing values or values outside the scale range
## (`geom_point()`).
```

```
## [1] "L14"
```

```
## Warning: Removed 4 rows containing missing values or values outside the scale range
## (`geom_point()`).
```

```
## Warning: Removed 4 rows containing missing values or values outside the scale range
## (`geom_point()`).
```

```
## [1] "L15"
```

```
## Warning: Removed 3 rows containing missing values or values outside the scale range
## (`geom_point()`).
```

```
## Warning: Removed 3 rows containing missing values or values outside the scale range
## (`geom_point()`).
```

```
## [1] "L16"
```

```
## Warning: Removed 2 rows containing missing values or values outside the scale range
## (`geom_point()`).
```

```
## Warning: Removed 2 rows containing missing values or values outside the scale range
## (`geom_point()`).
```

```
## [1] "L17"
```

```
## Warning: Removed 4 rows containing missing values or values outside the scale range
## (`geom_point()`).
```

```
## Warning: Removed 4 rows containing missing values or values outside the scale range
## (`geom_point()`).
```

```
## [1] "L18"
```

```
## Warning: Removed 2 rows containing missing values or values outside the scale range
## (`geom_point()`).
```

```
## Warning: Removed 2 rows containing missing values or values outside the scale range
## (`geom_point()`).
```

```
## [1] "L19"
```

```
## Warning: Removed 4 rows containing missing values or values outside the scale range
## (`geom_point()`).
```

```
## Warning: Removed 4 rows containing missing values or values outside the scale range
## (`geom_point()`).
```

```
## [1] "L20"
```

```
## Warning: Removed 1 row containing missing values or values outside the scale range
## (`geom_point()`).
```

```
## Warning: Removed 1 row containing missing values or values outside the scale range
## (`geom_point()`).
```

```
## [1] "L21"
```

```
## Warning: Removed 2 rows containing missing values or values outside the scale range
## (`geom_point()`).
```

```
## Warning: Removed 2 rows containing missing values or values outside the scale range
## (`geom_point()`).
```

```
## [1] "L22"
```

```
## Warning: Removed 2 rows containing missing values or values outside the scale range
## (`geom_point()`).
## Removed 2 rows containing missing values or values outside the scale range
## (`geom_point()`).
```

```
## [1] "L23"
```

```
## Warning: Removed 6 rows containing missing values or values outside the scale range
## (`geom_point()`).
```

```
## Warning: Removed 6 rows containing missing values or values outside the scale range
## (`geom_point()`).
```

```
## [1] "L24"
```

```
## Warning: Removed 2 rows containing missing values or values outside the scale range
## (`geom_point()`).
```

```
## Warning: Removed 2 rows containing missing values or values outside the scale range
## (`geom_point()`).
```

```
## [1] "L25"
```

```
## Warning: Removed 3 rows containing missing values or values outside the scale range
## (`geom_point()`).
```

```
## Warning: Removed 3 rows containing missing values or values outside the scale range
## (`geom_point()`).
```

```
## [1] "L26"
```

```
## Warning: Removed 2 rows containing missing values or values outside the scale range
## (`geom_point()`).
```

```
## Warning: Removed 2 rows containing missing values or values outside the scale range
## (`geom_point()`).
```

```
## [1] "L27"
```

```
## Warning: Removed 3 rows containing missing values or values outside the scale range
## (`geom_point()`).
```

```
## Warning: Removed 3 rows containing missing values or values outside the scale range
## (`geom_point()`).
```

```
## [1] "L28"
```

```
## Warning: Removed 1 row containing missing values or values outside the scale range
## (`geom_point()`).
```

```
## Warning: Removed 1 row containing missing values or values outside the scale range
## (`geom_point()`).
```

```
## [1] "L29"
```

```
## Warning: Removed 2 rows containing missing values or values outside the scale range
## (`geom_point()`).
```

```
## Warning: Removed 2 rows containing missing values or values outside the scale range
## (`geom_point()`).
```

```
## [1] "L3"
```

```
## Warning: Removed 3 rows containing missing values or values outside the scale range
## (`geom_point()`).
```

```
## Warning: Removed 3 rows containing missing values or values outside the scale range
## (`geom_point()`).
```

```
## [1] "L31"
```

```
## Warning: Removed 2 rows containing missing values or values outside the scale range
## (`geom_point()`).
```

```
## Warning: Removed 2 rows containing missing values or values outside the scale range
## (`geom_point()`).
```

```
## [1] "L32"
```

```
## Warning: Removed 3 rows containing missing values or values outside the scale range
## (`geom_point()`).
```

```
## Warning: Removed 3 rows containing missing values or values outside the scale range
## (`geom_point()`).
```

```
## [1] "L33"
```

```
## [1] "L34"
```

```
## Warning: Removed 5 rows containing missing values or values outside the scale range
## (`geom_point()`).
```

```
## Warning: Removed 5 rows containing missing values or values outside the scale range
## (`geom_point()`).
```

```
## [1] "L35"
```

```
## Warning: Removed 3 rows containing missing values or values outside the scale range
## (`geom_point()`).
```

```
## Warning: Removed 3 rows containing missing values or values outside the scale range
## (`geom_point()`).
```

```
## [1] "L36"
```

```
## Warning: Removed 3 rows containing missing values or values outside the scale range
## (`geom_point()`).
## Removed 3 rows containing missing values or values outside the scale range
## (`geom_point()`).
```

```
## [1] "L37"
```

```
## Warning: Removed 3 rows containing missing values or values outside the scale range
## (`geom_point()`).
## Removed 3 rows containing missing values or values outside the scale range
## (`geom_point()`).
```

```
## [1] "L38"
```

```
## [1] "L39"
```

```
## Warning: Removed 6 rows containing missing values or values outside the scale range
## (`geom_point()`).
```

```
## Warning: Removed 6 rows containing missing values or values outside the scale range
## (`geom_point()`).
```

```
## [1] "L40"
```

```
## Warning: Removed 3 rows containing missing values or values outside the scale range
## (`geom_point()`).
```

```
## Warning: Removed 3 rows containing missing values or values outside the scale range
## (`geom_point()`).
```

```
## [1] "L41"
```

```
## Warning: Removed 6 rows containing missing values or values outside the scale range
## (`geom_point()`).
```

```
## Warning: Removed 6 rows containing missing values or values outside the scale range
## (`geom_point()`).
```

```
## [1] "L5"
```

```
## Warning: Removed 3 rows containing missing values or values outside the scale range
## (`geom_point()`).
```

```
## Warning: Removed 3 rows containing missing values or values outside the scale range
## (`geom_point()`).
```

```
## [1] "L6"
```

```
## Warning: Removed 7 rows containing missing values or values outside the scale range
## (`geom_point()`).
```

```
## Warning: Removed 7 rows containing missing values or values outside the scale range
## (`geom_point()`).
```

```
## [1] "L7"
```

```
## Warning: Removed 2 rows containing missing values or values outside the scale range
## (`geom_point()`).
```

```
## Warning: Removed 2 rows containing missing values or values outside the scale range
## (`geom_point()`).
```

```
## [1] "L8"
```

```
## Warning: Removed 2 rows containing missing values or values outside the scale range
## (`geom_point()`).
## Removed 2 rows containing missing values or values outside the scale range
## (`geom_point()`).
```

```
## [1] "L9"
```

```
## Warning: Removed 2 rows containing missing values or values outside the scale range
## (`geom_point()`).
## Removed 2 rows containing missing values or values outside the scale range
## (`geom_point()`).
```

Linear model but plotting both cals and suspects

```
for(lab_this in levels(factor(data_all_SU$lab))) {
  print(lab_this)
    
  print(
    ggplot(data = data_all_SU_pred_lm %>%
             filter(lab == lab_this)) +
      geom_point(mapping = aes(x = logRF_pred,
                               y = logRF_SU,
                               color = type),
                 size = 3,
                 alpha = 0.8) +
      geom_abline(intercept = 0, slope = 1) +
      scale_x_continuous(limits = c(min_val, max_val)) +
      scale_y_continuous(limits = c(min_val, max_val)) +
      scale_color_manual(values = c("#659D8B", "#8C2155")) +
      labs(x = "linear fit", y = "orbitrap SU", title = lab_this) +
      my_theme +
      theme(axis.text.x = element_text(angle = 90))
  )
}
```

```
## [1] "L1"
```

```
## Warning: Removed 2 rows containing missing values or values outside the scale range
## (`geom_point()`).
```

```
## [1] "L10"
```

```
## Warning: Removed 12 rows containing missing values or values outside the scale range
## (`geom_point()`).
```

```
## [1] "L11"
```

```
## Warning: Removed 4 rows containing missing values or values outside the scale range
## (`geom_point()`).
```

```
## [1] "L12"
```

```
## Warning: Removed 1 row containing missing values or values outside the scale range
## (`geom_point()`).
```

```
## [1] "L14"
```

```
## Warning: Removed 8 rows containing missing values or values outside the scale range
## (`geom_point()`).
```

```
## [1] "L15"
```

```
## Warning: Removed 5 rows containing missing values or values outside the scale range
## (`geom_point()`).
```

```
## [1] "L16"
```

```
## Warning: Removed 5 rows containing missing values or values outside the scale range
## (`geom_point()`).
```

```
## [1] "L17"
```

```
## Warning: Removed 4 rows containing missing values or values outside the scale range
## (`geom_point()`).
```

```
## [1] "L18"
```

```
## Warning: Removed 4 rows containing missing values or values outside the scale range
## (`geom_point()`).
```

```
## [1] "L19"
```

```
## Warning: Removed 10 rows containing missing values or values outside the scale range
## (`geom_point()`).
```

```
## [1] "L20"
```

```
## Warning: Removed 2 rows containing missing values or values outside the scale range
## (`geom_point()`).
```

```
## [1] "L21"
```

```
## Warning: Removed 3 rows containing missing values or values outside the scale range
## (`geom_point()`).
```

```
## [1] "L22"
```

```
## Warning: Removed 3 rows containing missing values or values outside the scale range
## (`geom_point()`).
```

```
## [1] "L23"
```

```
## Warning: Removed 12 rows containing missing values or values outside the scale range
## (`geom_point()`).
```

```
## [1] "L24"
```

```
## Warning: Removed 6 rows containing missing values or values outside the scale range
## (`geom_point()`).
```

```
## [1] "L25"
```

```
## Warning: Removed 11 rows containing missing values or values outside the scale range
## (`geom_point()`).
```

```
## [1] "L26"
```

```
## Warning: Removed 4 rows containing missing values or values outside the scale range
## (`geom_point()`).
```

```
## [1] "L27"
```

```
## Warning: Removed 5 rows containing missing values or values outside the scale range
## (`geom_point()`).
```

```
## [1] "L28"
```

```
## Warning: Removed 2 rows containing missing values or values outside the scale range
## (`geom_point()`).
```

```
## [1] "L29"
```

```
## Warning: Removed 7 rows containing missing values or values outside the scale range
## (`geom_point()`).
```

```
## [1] "L3"
```

```
## Warning: Removed 4 rows containing missing values or values outside the scale range
## (`geom_point()`).
```

```
## [1] "L31"
```

```
## Warning: Removed 4 rows containing missing values or values outside the scale range
## (`geom_point()`).
```

```
## [1] "L32"
```

```
## Warning: Removed 8 rows containing missing values or values outside the scale range
## (`geom_point()`).
```

```
## [1] "L33"
```

```
## Warning: Removed 1 row containing missing values or values outside the scale range
## (`geom_point()`).
```

```
## [1] "L34"
```

```
## Warning: Removed 10 rows containing missing values or values outside the scale range
## (`geom_point()`).
```

```
## [1] "L35"
```

```
## Warning: Removed 5 rows containing missing values or values outside the scale range
## (`geom_point()`).
```

```
## [1] "L36"
```

```
## Warning: Removed 9 rows containing missing values or values outside the scale range
## (`geom_point()`).
```

```
## [1] "L37"
```

```
## Warning: Removed 5 rows containing missing values or values outside the scale range
## (`geom_point()`).
```

```
## [1] "L38"
```

```
## Warning: Removed 1 row containing missing values or values outside the scale range
## (`geom_point()`).
```

```
## [1] "L39"
```

```
## Warning: Removed 11 rows containing missing values or values outside the scale range
## (`geom_point()`).
```

```
## [1] "L40"
```

```
## Warning: Removed 6 rows containing missing values or values outside the scale range
## (`geom_point()`).
```

```
## [1] "L41"
```

```
## Warning: Removed 10 rows containing missing values or values outside the scale range
## (`geom_point()`).
```

```
## [1] "L5"
```

```
## Warning: Removed 6 rows containing missing values or values outside the scale range
## (`geom_point()`).
```

```
## [1] "L6"
```

```
## Warning: Removed 14 rows containing missing values or values outside the scale range
## (`geom_point()`).
```

```
## [1] "L7"
```

```
## Warning: Removed 3 rows containing missing values or values outside the scale range
## (`geom_point()`).
```

```
## [1] "L8"
```

```
## Warning: Removed 6 rows containing missing values or values outside the scale range
## (`geom_point()`).
```

```
## [1] "L9"
```

```
## Warning: Removed 3 rows containing missing values or values outside the scale range
## (`geom_point()`).
```

All linear model-projected datasets in one plot

```
ggplot(data = data_all_SU_pred_lm %>% 
         na.omit()) +
      geom_point(mapping = aes(x = logRF_pred,
                               y = logRF_SU,
                               color = type),
                 size = 2,
                 alpha = 0.7) +
      geom_abline(intercept = 0, slope = 1) +
      scale_x_continuous(limits = c(10, 16)) +
      scale_y_continuous(limits = c(10, 16)) +
      scale_color_manual(values = c("#659D8B", "#8C2155")) +
      facet_wrap(~ lab) +
      labs(x = "linear fit", y = "orbitrap SU", title = lab_this) +
      my_theme +
      theme(axis.text.x = element_text(angle = 90))
```

## logRF range

Calculating the spread of logRF for each lab, with original and LM
projected data

```
logRF_spread_lm = data_all_SU_pred_lm %>% 
  group_by(lab) %>% 
  summarize(RF_spread_orig = max(logRF) - min(logRF),
            RF_full_spread_orig = paste0(min(logRF), " - ", max(logRF)),
            RF_spread_lm = max(logRF_pred) - min(logRF_pred),
            RF_full_spread_lm = paste0(min(logRF_pred), " - ", max(logRF_pred))) %>% 
  ungroup() %>% 
  mutate(spread = case_when(RF_spread_orig > RF_spread_lm ~ "orig_spread_larger",
                            RF_spread_orig < RF_spread_lm ~ "lm_spread_larger",
                            TRUE ~ "NA"))
```

Visualize the distribution of logRFs

```
ggplot(data_all_SU_pred_lm) +
  geom_histogram(aes(x = logRF, y = after_stat(density)),
                 bins = 30, fill = "black", color = "white", alpha = 0.7) +
  geom_histogram(aes(x = logRF_pred, y = after_stat(density)),
                 bins = 30, fill = "black", color = "white", alpha = 0.7) +
  geom_density(aes(x = logRF),
               color = "#659D8B", linewidth = 1, fill = "#659D8B", alpha = 0.5) +
  geom_density(aes(x = logRF_pred),
               color = "#8C2155", linewidth = 1, fill = "#8C2155", alpha = 0.5) +
  facet_wrap(~ lab) +
  labs(x = "logRF", y = "Density") +
  my_theme
```

Visualize the ranges of projected and raw logRFs

```
spread_data = data_all_SU_pred_lm %>% 
  na.omit() %>% 
  group_by(lab) %>% 
  summarize(R2_original = cor(logRF, logRF_SU)^2,
            R2_lm = cor(logRF_pred, logRF_SU)^2,
            min_logRF = min(logRF),
            max_logRF = max(logRF),
            spread = max_logRF - min_logRF,
            min_logRF_lm = min(logRF_pred),
            max_logRF_lm = max(logRF_pred),
            spread_lm = max_logRF_lm - min_logRF_lm,
            spread_ratio = spread / spread_lm,
            spread_difference = spread - spread_lm) %>% 
  ungroup %>% 
  mutate(lab = recode(lab,
                      "L3" = "*L3", 
                      "L8" = "*L8", 
                      "L9" = "*L9", 
                      "L10" = "*L10",
                      "L17" = "*L17", 
                      "L27" = "*L27", 
                      "L28" = "*L28",
                      "L36" = "*L36", 
                      "L37" = "*L37",
                      "L41" = "*L41"))

fig_1 = ggplot(spread_data,
               aes(y = fct_reorder(lab, R2_lm))) +
  geom_linerange(aes(xmin = min_logRF,
                     xmax = max_logRF),
                 size = 2.5, color = "grey") +
  geom_point(aes(x = min_logRF),
             color = "grey50",
             size = 2.5) +
  geom_point(aes(x = max_logRF),
             color = "grey50",
             size = 2.5) +
  geom_linerange(aes(xmin = min_logRF_lm,
                     xmax = max_logRF_lm),
                 size = 2, color = "#8C2155", alpha = 0.8) +
  geom_point(aes(x = min_logRF_lm),
             color = "#531332",
             size = 2) +
  geom_point(aes(x = max_logRF_lm),
             color = "#531332",
             size = 2) +
  scale_x_continuous(breaks = c(10, 11, 12, 13, 14, 15, 16, 17, 18)) +
  labs(y = expression("Dataset (ordered by "~italic(R)^2~")"),
       x = "logRF") +
  theme_minimal() +
  theme(axis.title = element_text(family = font,
                                  size = 12,
                                  color = basecolor),
        axis.text = element_text(family = font,
                                 size = 10, 
                                 color = basecolor))
```

```
## Warning: Using `size` aesthetic for lines was deprecated in ggplot2 3.4.0.
## ℹ Please use `linewidth` instead.
## This warning is displayed once every 8 hours.
## Call `lifecycle::last_lifecycle_warnings()` to see where this warning was
## generated.
```

```
fig_1
```

Visualize the correlation of the range ratio (raw range/projected
range) and the Pearson R2

```
fig_S2 = ggplot() +
  geom_point(data = spread_data %>% 
               filter(lab != "L18",
                      lab != "*L28"),
               mapping = aes(x = R2_lm,
                           y = spread_ratio),
             color = "#262626",
             fill = "#262626",
             stroke = 1,
             alpha = 0.7,
             size = 3,
             shape = 21) +
  geom_point(data = spread_data %>%
               filter(lab %in% c("L18", "*L28")),
             mapping = aes(x = R2_lm,
                           y = spread_ratio),
             color = "#262626",
             fill = "#EFBDD5",
             stroke = 1,
             alpha = 0.7,
             size = 3, 
             shape = 21) +
  labs(y = "Range ratio", x = expression(""~italic(R)^2~" of the calibrants")) +
  my_theme +
  theme(aspect.ratio = 1,
        axis.title = element_text(family = font,
                                  size = 12,
                                  color = basecolor))

fig_S2
```

```
spread_data %>% 
  cor_test(R2_lm, spread_ratio, method = "spearman")
```

```
## # A tibble: 1 × 6
##   var1  var2           cor statistic      p method  
##   <chr> <chr>        <dbl>     <dbl>  <dbl> <chr>   
## 1 R2_lm spread_ratio -0.43     12068 0.0083 Spearman
```

## Statistical analysis

Calculating the statistics for logRF with and without projection with
a linear model

```
data_all_SU_summary_lm = data_all_SU_pred_lm %>%
  na.omit() %>% 
  group_by(lab, type) %>%
  summarize(RMSE_original = rmse(logRF, logRF_SU),
            RMSE_lm = rmse(logRF_pred, logRF_SU),
            MAD_original = mad(logRF, logRF_SU),
            MAD_lm = mad(logRF_pred, logRF_SU),
            Q95_original = quantile(abs(logRF - logRF_SU), probs = c(0.95)),
            Q95_lm = quantile(abs(logRF_pred - logRF_SU), probs = c(0.95)),
            R2_original = cor(logRF, logRF_SU)^2, 
            R2_lm = cor(logRF_pred, logRF_SU)^2) %>%
  ungroup()
```

```
## `summarise()` has grouped output by 'lab'. You can override using the `.groups`
## argument.
```

Here we are testing whether the RMSEs of suspects and calibrants are
statistically the same or if there is a statistical difference (using
F-test, LM-projected data)

```
results_var_test_lm = tibble()
for(lab_this in levels(factor(data_all_SU_pred_lm$lab))) {
    data_xy_this_that = data_all_SU_pred_lm %>%
      filter(lab == lab_this) 
    result = var.test(lm(logRF_SU ~ 0 + offset(1*logRF_pred), 
                         data = data_xy_this_that %>%
                           filter(type == "cals")), 
                      lm(logRF_SU ~ 0 + offset(1*logRF_pred), 
                         data = data_xy_this_that %>%
                           filter(type == "sus")))
    results_var_test_lm = results_var_test_lm %>%
      bind_rows(tibble(lab = lab_this,
                       p_value = result$p.value))
}
```

Four labs had statistically different RMSEs for calibrants and
suspects: L40, L1, L22 and L27. We will also plot them in an interactive
scatterplot.

```
excluded_labs <- c("L40", "L1", "L22", "L27")

data_rmse <- data_all_SU_pred_lm %>% 
  na.omit() %>% 
  group_by(type, lab) %>% 
  summarize(RMSE_lm = rmse(logRF_pred, logRF_SU)) %>% 
  ungroup() %>% 
  select(lab, type, RMSE_lm) %>% 
  spread(key = type, value = RMSE_lm) %>% 
  mutate(group = if_else(lab %in% excluded_labs, "Statistically different", "Not statistically different"))
```

```
## `summarise()` has grouped output by 'type'. You can override using the
## `.groups` argument.
```

```
p <- ggplot(data_rmse) +
  geom_point(mapping = aes(x = cals, 
                           y = sus, 
                           color = group, 
                           text = lab),
             size = 3,
             alpha = 0.8) +
  geom_abline(intercept = 0, slope = 1) +
  scale_color_manual(values = c("Not statistically different" = "grey30",
                                "Statistically different" = "#8C2155")) +
  labs(x = "RMSE cal", y = "RMSE sus", color = "RMSEs") +
  my_theme +
  theme(aspect.ratio = 1)

ggplotly(p, tooltip = "text")
```

```
p
```

Now let’s compare RMSEs of suspects from GAM- and lm-predicted
logRFs

```
# combining the GAM and lm data
data_all_SU_pred_gam_lm = data_all_SU_pred %>% 
  rename(logRF_gam = logRF_pred) %>% 
  left_join(data_all_SU_pred_lm %>% 
              rename(logRF_lm = logRF_pred)
            )
```

```
## Joining with `by = join_by(lab, compound, logRF, logRF_SU, type, RT)`
```

```
results_var_test_gam_lm = tibble()
for(lab_this in levels(factor(data_all_SU_pred_gam_lm$lab))) {
    data_xy_this_that = data_all_SU_pred_gam_lm %>%
      filter(lab == lab_this,
             type == "sus",
             lab != "L38") #remove anchoring lab from statistical analysis
    lm_model = lm(logRF_SU ~ 0 + offset(1*logRF_lm),
                  data = data_xy_this_that)
    gam_model = lm(logRF_SU ~ 0 + offset(1*logRF_gam),
                   data = data_xy_this_that)
    result = var.test(lm(logRF_SU ~ 0 + offset(1*logRF_gam),
                         data = data_xy_this_that),
                      lm(logRF_SU ~ 0 + offset(1*logRF_lm),
                         data = data_xy_this_that))
    n = n_distinct(data_xy_this_that$logRF_SU,
                   na.rm = T)
    residuals_gam = residuals(lm(logRF_SU ~ 0 + offset(1*logRF_gam),
                                 data = data_xy_this_that))
    residuals_lm = residuals(lm(logRF_SU ~ 0 + offset(1*logRF_lm),
                                data = data_xy_this_that))
    variance_gam = (sum(residuals_gam^2))/n
    variance_lm = (sum(residuals_lm^2))/n
    results_var_test_gam_lm = results_var_test_gam_lm %>%
      bind_rows(tibble(lab = lab_this,
                       p_value = result$p.value,
                       variance_gam = variance_gam,
                       variance_lm = variance_lm))
}
```

```
## Warning in qf(1 - BETA, DF.x, DF.y): NaNs produced
```

```
## Warning in qf(BETA, DF.x, DF.y): NaNs produced
```

From this, we could see that for almost all datasets, there were no
statistical difference between using a linear model and GAM for
transferring the logRF values to the same scale. Only for 1 lab (L20), p
< 0.05, indicating statistical significance. Looking further, we
could see that the variance was lower for that lab when using the linear
model, but also overall generally providing lower variance. This
indicate that GAM may overfit the data, or that GAM was too flexible.
Thus, we decided to go further with linearly fitting the logRFs from the
different labs to the scale of L38.

## Anchoring logRF values for lineplot

For the linegraph, we want to see essentially whether there are any
specific chemicals which vary more with the logRF (or predicted/anchored
logRF). Therefore, for this we will redo the linear regression but
instead of just using the cals we will now use all chemicals (cals +
sus).

```
data_all_SU_pred_lm_linegraph = tibble()

for(lab_this in levels(factor(data_all_SU$lab))) {
  print(lab_this)
  data_xy_this_that = data_all_SU %>%
    filter(lab == lab_this) %>%
    select(lab, compound, logRF, logRF_SU, type) %>%
    unique()

    model_this_lm = lm(logRF_SU ~ logRF,
                       data = data_xy_this_that) 

    data_xy_this_that = data_xy_this_that %>%
      mutate(logRF_pred = predict(model_this_lm,
                                  newdata = data_xy_this_that))

    data_all_SU_pred_lm_linegraph = data_all_SU_pred_lm_linegraph %>%
      bind_rows(data_xy_this_that)
}
```

```
## [1] "L1"
## [1] "L10"
## [1] "L11"
## [1] "L12"
## [1] "L14"
## [1] "L15"
## [1] "L16"
## [1] "L17"
## [1] "L18"
## [1] "L19"
## [1] "L20"
## [1] "L21"
## [1] "L22"
## [1] "L23"
## [1] "L24"
## [1] "L25"
## [1] "L26"
## [1] "L27"
## [1] "L28"
## [1] "L29"
## [1] "L3"
## [1] "L31"
## [1] "L32"
## [1] "L33"
## [1] "L34"
## [1] "L35"
## [1] "L36"
## [1] "L37"
## [1] "L38"
## [1] "L39"
## [1] "L40"
## [1] "L41"
## [1] "L5"
## [1] "L6"
## [1] "L7"
## [1] "L8"
## [1] "L9"
```
