# Supplementary material for: In-Depth Analysis of the Data from an Interlaboratory Study of Quantitative Non-Target Screening—How Do the Instrumental Methods Compare?
Source: Molecules. 2026 Mar 6;31(5):875. doi: 10.3390/molecules31050875 (PMC12986245; doi:10.3390/molecules31050875)
Supplement: Supplementary file 1 [file molecules-31-00875-s001.zip › Code S4_sum_squares_calculations.html]

Calculating the sum of squares for chemicals and datasets


# Calculating the sum of squares for chemicals and datasets

#### Louise Malm

## Read in the libraries

```
library(tidyverse)
library(rstatix)
library(plotly)
source("theme.R")
```

## Read in the data

Experimental and projected RF data from the interlaboratory
comparison.

```
RF_data = read_delim("pred_logRF.csv",
                          delim = ",",
                          col_names = T)
```

## Calculating sum of squares

Calculate mean(logRF), n, and SS for each row (= each chemical)

```
RF_lm_wide <- RF_data %>% 
  select(lab, compound, logRF_lm_line) %>% 
  pivot_wider(names_from = lab,
              values_from = logRF_lm_line)

RF_lm_wide <- RF_lm_wide %>% 
  mutate(mean_logRF = rowMeans(across(-compound),
                               na.rm = T),
         n_cols = rowSums(!is.na(across(-c(compound, mean_logRF))))) %>% 
  rowwise() %>% 
  mutate(ss_chemical = sum((c_across(-c(compound, mean_logRF, n_cols)) - mean_logRF)^2, na.rm = T) / n_cols) %>% 
  ungroup()
```

Calculate n and SS for each column (= each dataset)

```
RF_lm_long <- RF_lm_wide %>% 
  pivot_longer(cols = -c(compound, mean_logRF, n_cols, ss_chemical),
               names_to = "lab",
               values_to = "logRF_lm_line")

ss_lab <- RF_lm_long %>% 
  filter(!is.na(logRF_lm_line)) %>% 
  group_by(lab) %>% 
  summarise(
    n_rows = n(),
    ss_lab = sum((logRF_lm_line - mean_logRF)^2) / n_rows) %>% 
  ungroup()
```

Combine into one dataframe

```
RF_ss_lm <- RF_lm_long %>% 
  na.omit() %>% 
  group_by(lab) %>% 
  left_join(ss_lab) %>% 
  ungroup() %>% 
  group_by(compound) %>% 
  left_join(RF_data) %>% 
  ungroup() %>% 
  select(lab, compound, type, mean_logRF, logRF_lm_line, n_cols, ss_chemical, n_rows, ss_lab)
```

Visualize SS chem vs average projected logRF

```
p <- ggplot(RF_ss_lm) +
  geom_point(mapping = aes(x = ss_chemical,
                           y = mean_logRF,
                           text = compound),
             color = "#8C2155",
             size = 3,
             alpha = 0.5) +
  labs(x = "Sum of squares, chemicals",
       y = "Average logRF") +
  theme_classic() +
  theme(aspect.ratio = 1)
```

```
## Warning in geom_point(mapping = aes(x = ss_chemical, y = mean_logRF, text =
## compound), : Ignoring unknown aesthetics: text
```

```
ggplotly(p, tooltip = "text")
```

```
## Warning: Aspect ratios aren't yet implemented, but you can manually set a
## suitable height/width
```

```
## Warning: Aspect ratios aren't yet implemented, but you can manually set a
## suitable height/width
```

```
RF_ss_figS4 <- RF_ss_lm %>% 
  select(compound, type, mean_logRF, n_cols, ss_chemical) %>% 
  unique()

fig_S4 <- ggplot() +
  geom_point(data = RF_ss_figS4 %>%
               filter(n_cols < 10),
             mapping = aes(x = ss_chemical,
                           y = mean_logRF),
             color = "#262626",
             fill = "#EFBDD5",
             alpha = 0.7,
             stroke = 1,
             shape = 21,
             size = 3,
             alpha = 0.7) +
  geom_point(data = RF_ss_figS4 %>%
               filter(n_cols >= 10),
             mapping = aes(x = ss_chemical,
                           y = mean_logRF),
             color = "#262626",
             fill = "#262626",
             alpha = 0.7,
             stroke = 1,
             size = 3,
             shape = 21) +
  labs(x = "Sum of squares, chemicals",
       y = "Average projected logRF") +
  my_theme +
  theme(aspect.ratio = 1,
        axis.title = element_text(family = font,
                                  size = 12,
                                  color = basecolor))
```

```
## Warning: Duplicated aesthetics after name standardisation: alpha
```

```
fig_S4
```

Spearman correlation of average logRF and SS chemical

```
RF_ss_lm %>% 
  cor_test(ss_chemical, mean_logRF, method = "spearman")
```

```
## # A tibble: 1 × 6
##   var1        var2         cor   statistic      p method  
##   <chr>       <chr>      <dbl>       <dbl>  <dbl> <chr>   
## 1 ss_chemical mean_logRF  -0.5 2194352036. 2e-129 Spearman
```
