# Supplementary material for: In-Depth Analysis of the Data from an Interlaboratory Study of Quantitative Non-Target Screening—How Do the Instrumental Methods Compare?
Source: Molecules. 2026 Mar 6;31(5):875. doi: 10.3390/molecules31050875 (PMC12986245; doi:10.3390/molecules31050875)
Supplement: Supplementary file 1 [file molecules-31-00875-s001.zip › Code S5_RF_clustering_analysis.html]

RF clustering analysis


# RF clustering analysis

#### Louise Malm & Anneli Kruve

## Read in the libraries

```
library(tidyverse)
source("theme.R")
```

## Read in the data

Experimental and projected RF data from the interlaboratory
comparison.

```
predRF_data = read_delim("pred_logRF_gam_lm.csv",
                          delim = ",",
                          col_names = T)
```

## Correlation matrices

Create the correlation matrices needed for heatmap and clustering

```
predRF_corr = predRF_data %>%
  select(Lab, Compound, `logRF LM`) %>%
  left_join(predRF_data %>%
              select(Lab, Compound, `logRF LM`),
            by = "Compound")
```

```
## Warning in left_join(., predRF_data %>% select(Lab, Compound, `logRF LM`), : Detected an unexpected many-to-many relationship between `x` and `y`.
## ℹ Row 1 of `x` matches multiple rows in `y`.
## ℹ Row 1 of `y` matches multiple rows in `x`.
## ℹ If a many-to-many relationship is expected, set `relationship =
##   "many-to-many"` to silence this warning.
```

## Clustering based on Pearson correlation

Pearson correlation data

```
predRF_data_heatmap_pear = predRF_corr %>%
  group_by(Lab.x, Lab.y) %>%
  summarise(similarity = 1-cor(`logRF LM.x`, `logRF LM.y`, method = "pearson")^2) %>%
  ungroup()

predRF_data_heatmap_pear = predRF_data_heatmap_pear %>% 
  pivot_wider(names_from = Lab.y,
              values_from = similarity)
```

Heatmap with Pearson correlation

```
heatmap(predRF_data_heatmap_pear %>% 
          column_to_rownames(var = "Lab.x") %>% 
          as.matrix(),
        scale = "none",
        col = colorRampPalette(c("#841F50", "#F7DEEA"))(256))
```

## Clustering based on Spearman correlation

Spearman correlation data

```
predRF_data_heatmap_spear = predRF_corr %>%
  group_by(Lab.x, Lab.y) %>%
  summarise(similarity = 1-cor(`logRF LM.x`, `logRF LM.y`, method = "spearman")^2) %>%
  ungroup()

predRF_data_heatmap_spear = predRF_data_heatmap_spear %>% 
  pivot_wider(names_from = Lab.y,
              values_from = similarity)
```

Heatmap with Spearman correlation

```
heatmap(predRF_data_heatmap_spear %>% 
          column_to_rownames(var = "Lab.x") %>% 
          as.matrix(),
        scale = "none",
        col = colorRampPalette(c("#841F50", "#F7DEEA"))(256))
```

## Agreement of Pearson and Spearman correlation

```
pearson = predRF_corr %>% 
  group_by(Lab.x, Lab.y) %>% 
  summarize(R2 = cor(`logRF LM.x`, `logRF LM.y`, method = "pearson")^2) %>% 
  ungroup() %>% 
  filter(Lab.x != Lab.y) %>%
  mutate(dataset_x = Lab.x,
         dataset_y = Lab.y) %>% 
  unite(combined, Lab.x, Lab.y) %>%
  rename(pearson = R2)

spearman = predRF_corr %>% 
  group_by(Lab.x, Lab.y) %>% 
  summarize(R2 = cor(`logRF LM.x`, `logRF LM.y`, method = "spearman")^2) %>% 
  ungroup() %>% 
  filter(Lab.x != Lab.y) %>% 
  mutate(dataset_x = Lab.x,
         dataset_y = Lab.y) %>% 
  unite(combined, Lab.x, Lab.y) %>% 
  rename(spearman = R2)

pearson_spearman = pearson %>% 
  left_join(spearman)

ggplot(data = pearson_spearman) +
  geom_point(mapping = aes(x = pearson,
                           y = spearman),
             color = "#8C2155",
             size = 3,
             alpha = 0.6) +
  xlab("Pearson R2") +
  ylab("Spearman R2") +
  my_theme +
  theme(aspect.ratio = 1)
```
