# Supplementary material for: In-Depth Analysis of the Data from an Interlaboratory Study of Quantitative Non-Target Screening—How Do the Instrumental Methods Compare?
Source: Molecules. 2026 Mar 6;31(5):875. doi: 10.3390/molecules31050875 (PMC12986245; doi:10.3390/molecules31050875)
Supplement: Supplementary file 1 [file molecules-31-00875-s001.zip › molecules-4147114_SI_text_figures_revised.pdf]

In-Depth Analysis of the Data from an Interlaboratory Study of Quantitative Non-Target Screening—How Do the Instrumental Methods Compare?

Louise Malm [1], Nikiforos Alygizakis [2,3], Reza Aalizadeh [4] and Anneli Kruve [1,5,*]

[1] Department of Chemistry, Stockholm University, Svante Arrhenius Väg 16, 114 18 Stockholm, Sweden; louise.malm@su.se

[2] Laboratory of Analytical Chemistry, Department of Chemistry, National and Kapodistrian University of Athens, Panepistimiopolis Zografou, 157 71 Athens, Greece; nalygizakis@chem.uoa.gr

[3] Environmental Institute, Okružná 784/42, 972 41 Koš, Slovakia

[4] Department of Environmental Health Sciences, Yale School of Public Health, Yale University, 60 College St., New Haven, CT 065 10, USA; reza.aalizadeh@yale.edu

[5] Department of Environmental Science, Stockholm University, Svante Arrhenius Väg 8, 114 18 Stockholm, Sweden

* Correspondence: anneli.kruve@su.se

Table of contents

Texts

SI S1	Motivation for removing compounds only detected in either low or high concentration spiked samples	3
SI S2	Comparison of the impact of including or removing compounds only detected in low or high concentration spiked samples	4
SI S3	Comparison of the impact of accounting for the dilution factor or not of one dataset on the overall reported results from the interlaboratory study	6

Figures

Fig. S1a	Comparison of errors from two methods using same instrument but different LC parameters	7
Fig. S1b	Comparison of errors from two methods using the same instrument and instrumental method but different acquisition methods	7
Fig. S2	Correlation of range ratio (raw logRF range / projected logRF range) and R^2 (of projected logRF compared to anchoring dataset R^2)	8
Fig. S3a	Heatmap with hierarchical clustering based on Pearson correlation	9
Fig. S3b	Heatmap with hierarchical clustering based on Spearman correlation	9
Fig S4	Correlation of SS_{chem} and average projected logRF	10

SI S1

While reanalyzing the data, a minor mistake related to the processing of reprocessed peaks in the previous study was observed. Namely, when filtering based on peak area ratios, we aimed to remove the compounds which had ratio below 5 or above 20. In addition, we also wanted to remove compounds that were only detected in the low concentrated sample in a given matrix. However, instead the compounds which were only detected in high concentrated samples were removed from the data, and those only detected in low concentration was kept. For this study, we decided to also remove the compounds detected in only low concentration samples, and keep the rest as is. I.e., the reprocessed results should contain only compounds with a peak area ratio between 5 and 20, (only compounds detected in both high and low concentrations in a given matrix). The motivation for this decision is as follows:

- Peak area ratio should theoretically be 10, thus removing those that have more than halved or doubled ratio makes sense as these might be incorrectly assigned peaks.
- Compounds detected only in low concentration are illogical since they should also be detectable at higher concentration with the same method and LC/MS system.
- Compounds detected only in high concentration might be logical, for example if the low concentration was below the limit of detection. On the other hand, we cannot be sure about these peak areas/retention times, thus these peaks were also removed.

SI S2

No differences were observed by visual inspection of boxplots displaying prediction errors when the compounds detected only in low concentrated samples were removed compared to when they were kept. However, minor numerical changes were observed, e.g., when looking at the outliers or the prediction error statistics. Comparing the top five most occurring outliers, we observed that the number of occurrences and number of laboratories in which they were considered an outlier differed between the original data (when the compounds only detected in low concentrated samples were kept, denoted as *Original*) and the updated data (when the compounds only detected in low concentrated samples were removed, denoted as *Updated*). The numbers are displayed in Tables SI-S2.1 – SI-S2.2, with the changing numbers displayed in red. The results showed that even though the number slightly changed by removing the compounds only detected in low concentrated samples, the order was still the same independent on if we look at the number of occurrences or in how many labs the outliers were found.

Table SI-S2.1. The number of times the top five most occurring outliers were considered an outlier across all samples, approaches and datasets when the compounds detected only in low concentrated samples were kept vs when they were removed.

**Compound**	**Number of occurrences**	
	**Original**	**Updated**
Monuron	84 occurrences	82 occurrences
2-methylbenzothiazole	50 occurrences	49 occurrences
Methidathion	48 occurrences	46 occurrences
Atrazine-desethyl-desisopropyl	45 occurrences	42 occurrences
2-hydroxybenzothiazole	44 occurrences	42 occurrences

Table SI-S2.2. The number of datasets in which the top five most occurring outliers were considered an outlier across all samples and approaches when the compounds detected only in low concentrated samples were kept vs when they were removed.

**Compound**	**Number of datasets (% of total in brackets)**	
	**Original**	**Updated**
Monuron	21 (55%)	20 (53%)
Atrazine-desethyl-2-hydroxy	16 (42%)	16 (42%)
Atrazine-desethyl-desisopropyl	16 (42%)	15 (39%)
2-methylbenzothiazole	15 (39%)	14 (37%)
Methidathion	14 (37%)	14 (37%)
Methomyl*	14 (37%)	NA

*Was considered an outlier in the same number of datasets as methidathion in original study

Comparing the error statistics (mean, median, max & 95% quantile fold error, and % less than 10× error) across all labs and samples, resulted in slightly deviating numbers if the compounds detected in only low concentrated samples were removed or kept (see Table SI-S2.3). Still, these differences were minor and practically insignificant, and moreover, it had no effect on the conclusions drawn in the 2024 publication. [1]

Table SI-S2.3. The difference in error statistics when the compounds detected only in low concentrated samples were kept vs when they were removed.

<table>
<tr><th rowspan="2">Approach</th><th colspan="2">Mean error</th><th colspan="2">Median error</th><th colspan="2">Max error</th><th colspan="2">95% quantile error</th><th colspan="2">% less than 10× error</th></tr>
<tr><th>Original</th><th>Updated</th><th>Original</th><th>Updated</th><th>Original</th><th>Updated</th><th>Original</th><th>Updated</th><th>Original</th><th>Updated</th></tr>
<tr><td>Parent - TP</td><td colspan="2">13×</td><td colspan="2">3.4×</td><td colspan="2">1 200×</td><td>44×</td><td>43×</td><td colspan="2">75.5%</td></tr>
<tr><td>Structurally similar</td><td colspan="2">17×</td><td colspan="2">3.4×</td><td colspan="2">2 850×</td><td colspan="2">53×</td><td colspan="2">75.5%</td></tr>
<tr><td>Close eluting</td><td>154×</td><td>156×</td><td colspan="2">5.0×</td><td colspan="2">66 500×</td><td>183×</td><td>180×</td><td>65.1%</td><td>65.0%</td></tr>
<tr><td>RandFor-IE</td><td colspan="2">5.5×</td><td colspan="2">2.4×</td><td colspan="2">797×</td><td>17×</td><td>16×</td><td>91.2%</td><td>91.3%</td></tr>
<tr><td>MLR-IE</td><td colspan="2">11×</td><td colspan="2">2.8×</td><td colspan="2">1 970×</td><td colspan="2">32×</td><td colspan="2">83.4%</td></tr>
</table>

SI S3

One set of results were from analysis of 10× diluted samples, however, originally this dilution was not accounted for when calculating the prediction errors. Thus, the reported concentrations for this dataset were around 10× too low, causing a higher prediction error. This was not a problem for the MLR-*IE* approach since this approach used the normalized peak areas (to atrazine-d_5) to assess the concentration. Neither was it a problem for the reprocessed results since these too were based on peak areas normalized to atrazine-d_5. Thus, only in the reported results for the parent-TP, structurally similar, close eluting and RandFor-*IE* approaches this influenced the perceived prediction error.

To account for the dilution factor, the predicted concentrations for the four abovementioned approaches were multiplied with 10. The impact of accounting for the dilution factor or not was assessed by visually comparing the boxplots (only high concentrated HPLC water) and the summary statistics. From the boxplot, it was observed that the boxes for the specific lab were lowered when accounting for the dilution factor, however, it had no effect on the overall conclusions. For the summary statistics (see Table SI-S3.1), although they slightly changed towards lower error, the changes were minor and had no effect on the general conclusions drawn in the original study. [1]

Table SI-S3.1. The difference in error statistics over all compounds, samples and datasets when accounting for the dilution factor (denoted *Updated*) vs when not accounting for the dilution factor (denoted *Original*).

**Approach**	**Mean error**		**Median error**		**Max error**		**95% quantile error**		**% less than 10× error**	
	**Original**	**Updated**	**Original**	**Updated**	**Original**	**Updated**	**Original**	**Updated**	**Original**	**Updated**
Parent - TP	140×	139×	3.9×	3.8×	368 000×		79×	73×	71.8%	73.1%
Structurally similar	99×		4.0×	3.8×	297 000×		108×	104×	70.4%	71.8%
Close eluting	1 180×		6.0×	5.9×	1 139 000×		573×	571×	60.3%	60.9%
RandFor-*IE*	15×	14×	3.0×		8 400×		28×		83.9%	84.3%
MLR-*IE*	2 960×		3.6×		1 175 000×		502×		75.5%	

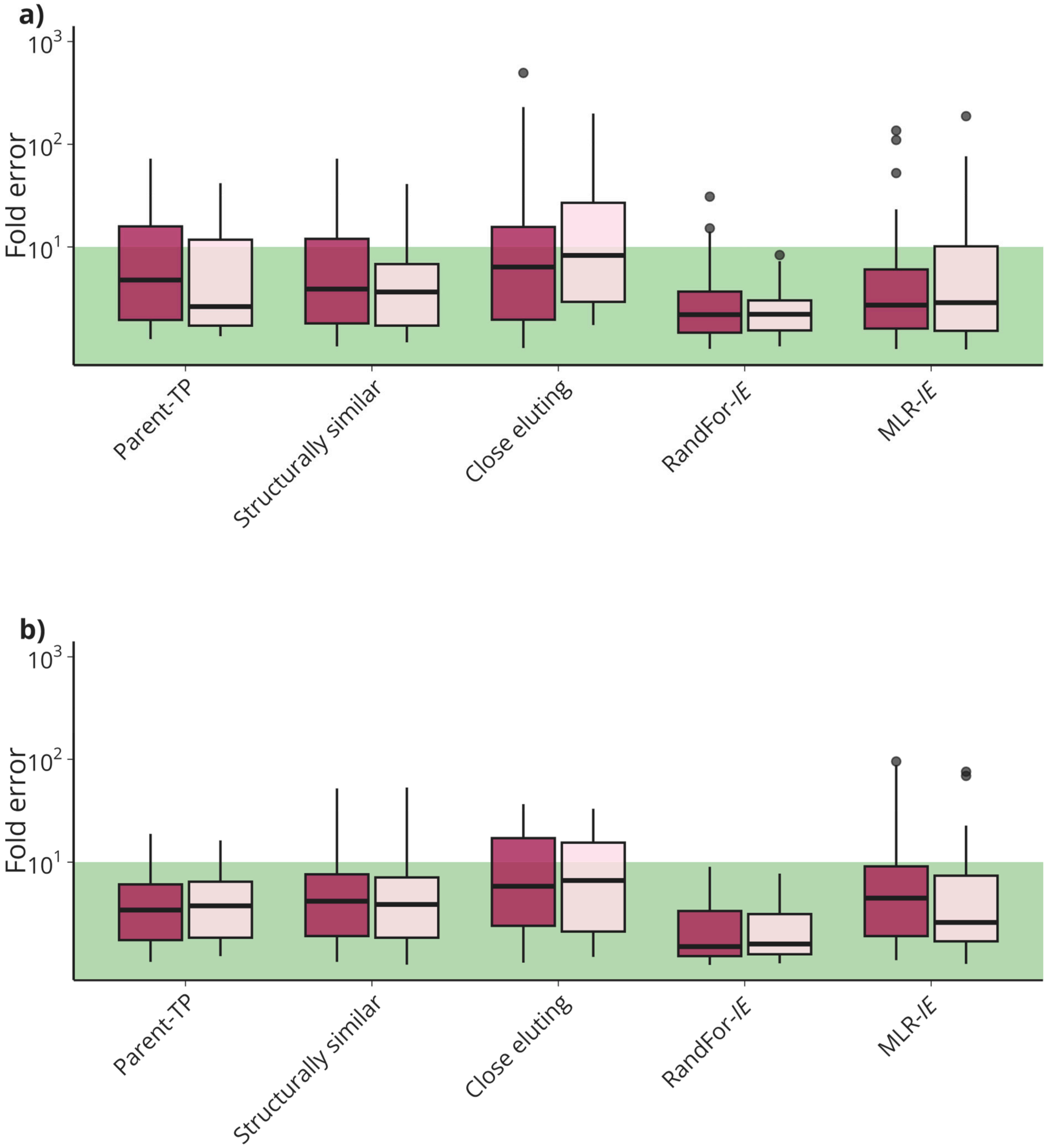

Figure S1. a) Datasets using same instruments but different LC parameters, and **b)** datasets using same instrument and instrumental method but different acquisition methods.

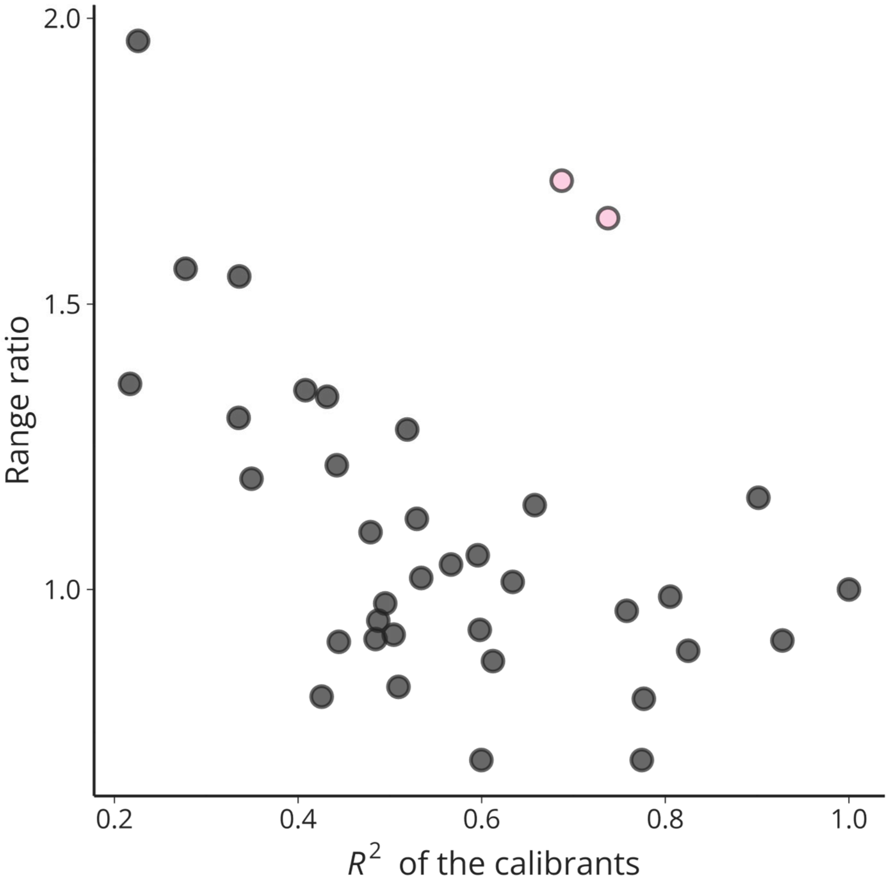

Figure S2. A weak correlation between range ratio (raw logRF range/projected logRF range) and R^2 of the calibrants was observed (Spearman correlation: -0.43, $p<0.05$). The two pink dots (light color) are the datapoints for L18 and L28, which had both high range ratio and high R^2.

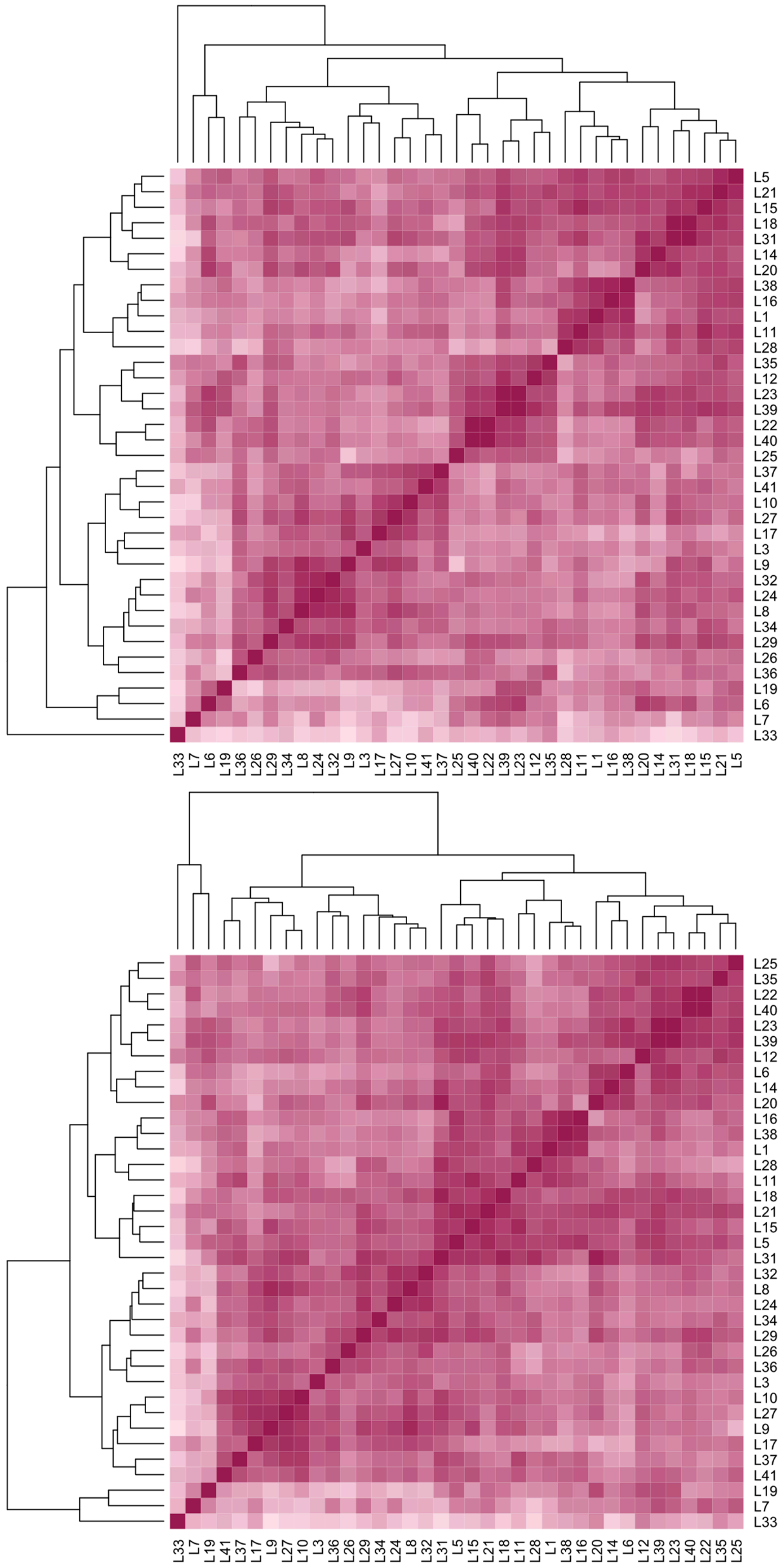

Figure S3. Heatmaps with hierarchical clustering based on **a)** Pearson correlation and **b)** Spearman correlation. Darker color indicated higher correlation coefficient while lighter color indicates lower correlation coefficient (Pearson and Spearman correlation coefficient, respectively).

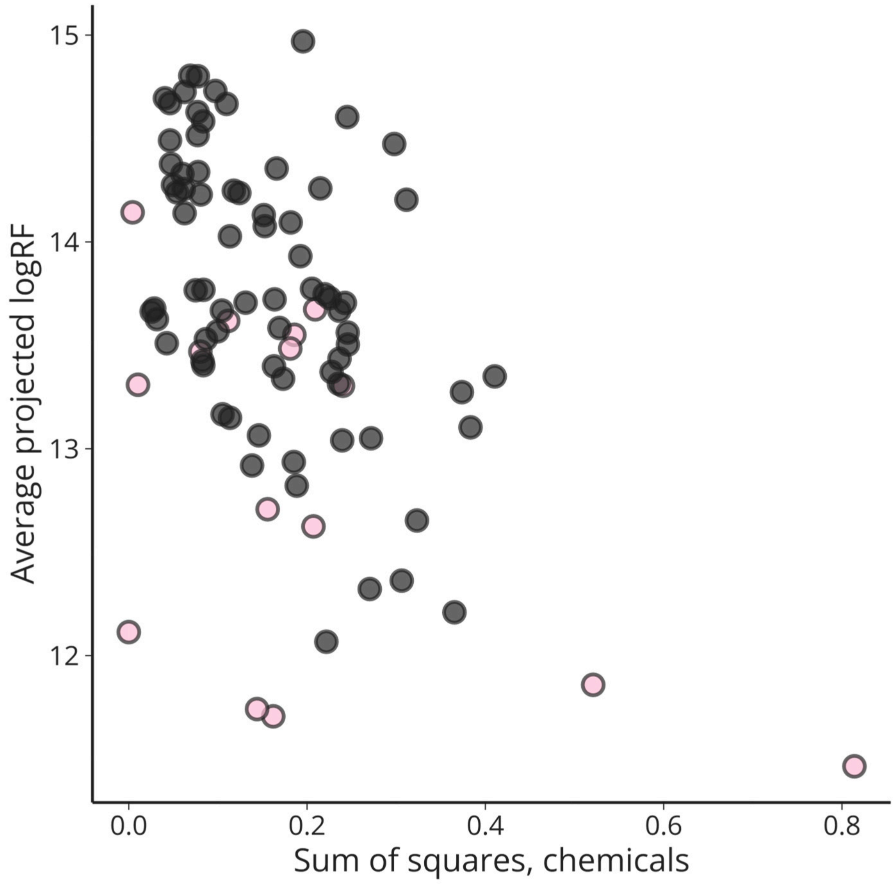

Figure S4. A weak correlation between average projected logRF and SS_{chem} was observed (Spearman correlation: -0.5, $p<<0.05$). Pink dots (light color) means that the SS comes from less than 10 datasets, grey dots (dark color) when SS comes from 10 or more datasets.

References

1. Malm, L.; Liigand, J.; Aalizadeh, R.; Alygizakis, N.; Ng, K.; Frøkjær, E.E.; Nanusha, M.Y.; Hansen, M.; Plassmann, M.; Bieber, S.; et al. Quantification Approaches in Non-Target LC/ESI/HRMS Analysis: An Interlaboratory Comparison. *Anal. Chem.* **2024**, *96*, 16215–16226, doi:10.1021/acs.analchem.4c02902.
